# Early pregnancy hyperglycaemia among pregnant women with risk factors for gestational diabetes increases the risk of pregnancy complications

**DOI:** 10.1038/s41598-024-76497-5

**Published:** 2024-10-24

**Authors:** Ka Wang Cheung, Tiffany Sin-Tung Au, Tat On Chan, Po Lam So, Felix Chi-Kin Wong, Mimi Tin Yan Seto

**Affiliations:** 1 Department of Obstetrics and Gynaecology, Queen Mary Hospital, The University of Hong Kong, 6/F, Professorial Block, 102 Pokfulam Road, Hong Kong, China; 2 Department of Obstetrics and Gynaecology, Tuen Mun Hospital, Tuen Mun, Hong Kong SAR China; 3 Department of Pathology, Queen Mary Hospital, Hong Kong, China

**Keywords:** Complication, Early onset, Diabetes, Diagnosis, Gestational, Management, Diabetes, Pre-diabetes

## Abstract

The current diagnostic criteria for gestational diabetes (GDM) were based on data from women after 24 weeks of gestation, but this may not be equally applicable for earlier gestation. There is insufficient data regarding early pregnancy glycaemia and the associated complications. We retrospectively reviewed 39,483 deliveries and 12,918 with risk factors for GDM underwent oral glucose tolerance test (OGTT) before 24 weeks of gestation. A strong and progressively positive association was observed with any pregnancy complications in both fasting glucose (FG) and 2 h glucose (2hG). The increased risk of developing any pregnancy complications started from FG 4.5–4.7 mmol/L and 2hG of 6.2–6.9mmol/L. Every increase by 1 mmol/L in FG or 2 hG levels increased the risk of developing any complications (aOR 1.614 for FG and 1.131 for 2hG), pre-eclampsia (aOR 1.472 for FG and 1.143 for 2hG), maternal insulin use (aOR 12.821 for FG and 2.366 for 2hG), primary Caesarean section(aOR 1.274 for FG and 1.099 for 2hG), shoulder dystocia (aOR 1.941 for FG and 1.282 for 2hG), macrosomia(aOR 2.203 for FG and 1.072 for 2hG), and large for gestation age(aOR 2.157 for FG and 1.074 for 2hG). Therefore, glycaemic levels in early pregnancy among high-risk women positively associated with pregnancy complications, even at levels below the current recommended diagnostic criteria for GDM.

**Supplementary Information:**

The online version contains supplementary material available at 10.1038/s41598-024-76497-5.

## Introduction

Glucose metabolism is regulated during pregnancy to provide adequate fetal development and maternal nutrition^1^. Gestational diabetes mellitus (GDM) is defined as hyperglycaemia first detected during pregnancy and the diagnostic criteria to identify ‘at risk’ women has been revised several times^2^. The original diagnostic criteria of GDM proposed by Dr. John B. O’Sullivan aimed to identify women at risk of diabetes development after delivery^3^. Later, it was found that women with GDM had a significant risk of adverse maternal and neonatal complications, and interventions to these women reduced the risk of large for gestational age fetus, macrosomia, shoulder dystocia and hypertensive disorders in pregnancy^4,5^. The objective of detecting women with GDM then shifted to antenatal hyperglyacemic treatment to prevent adverse perinatal outcomes. The diagnostic criteria of GDM was subsequently revised following the Hyperglycaemia and Adverse Pregnancy Outcomes (HAPO) study, which found a strong and continuous rise in the risk of developing pregnancy complications with increasing maternal glucose levels less severe than overt diabetes^6^. However, the lack of a definitive threshold to dichotomize women at risk of hyperglycaemic complications resulted in the proposed diagnostic values for GDM, based on a consensus of identifying women at 1.75 odd to deliver a newborn with complications above mean glucose values^7^. This diagnostic criteria was endorsed by World Health Organization (WHO) in 2013 and widely practiced clinically^2^. However, women below this diagnostic cut-off of GDM were still at risk of hyperglycaemic pregnancy complications^8^.

Although the HAPO study was carried out in pregnant women between 24 and 32 weeks of gestation, WHO recommended utilizing the same diagnostic criteria for earlier gestations (early onset GDM)^2^. A meta-analysis of 13 cohort studies suggested that early onset GDM increased the risk of pregnancy complications despite treatment^9^. However, 60% of women with a fasting plasma glucose (FG) ≥ 5.1 mmol/L at early gestation would not meet the diagnostic criteria of GDM when oral glucose tolerance test (OGTT) was repeated at 24–28 weeks of gestation^10^. In a randomized controlled trial, one-third of women diagnosed with early onset GDM recovered without any intervention^11^. Previous studies could not demonstrate beneficial effects from earlier screening and treatment for early onset GDM^12,13^. These highlighted the limitation of the current diagnostic criteria in early gestation to identify women who would require interventions for hyperglycaemia to prevent maternal and perinatal complications^14^. There was insufficient data regarding early pregnancy glycaemia and the associated complications. The aim of this study was to evaluate the incidence of different pregnancy complications with various FG and 2-hour plasma glucose (2hG) levels before 24 weeks of gestation.

## Methods

This was a retrospective study conducted at Queen Mary Hospital (QMH), a university affiliated hospital in Hong Kong, between 2010 and 2019. Basic demographics and clinical details were collected via the Clinical Data Analysis and Reporting System (CDARS), a well-developed electronic database developed by the Hospital Authority, Hong Kong to record clinical data in public settings. At QMH, all women attending antenatal visits were routinely screened for risk factors of GDM at their first booking visit, which included advanced maternal age (≥ 35 years old), obesity with body mass index (BMI) ≥ 25 kg/m^2^, polycystic ovarian syndrome, multiple pregnancy, polyhydramnios, glycosuria, personal history of unexplained stillbirth, macrosomia (≥ 4000 g) or GDM in previous pregnancy, and family history of diabetes mellitus. Women with any of the above risk factors underwent an 75-g OGTT before 24 weeks of gestation. Venous plasma samples were collected to determine the FG and 2hG level. Women with multiple pregnancy were excluded in this study. For women with more than one pregnant event during the studied period, only the latest pregnancy was included in the analysis to avoid duplication.

Terminology of hyperglycaemia first detected by OGTT during pregnancy was classified as either diabetes in pregnancy (DIP) or GDM following WHO recommendation. WHO recommended a change in the diagnostic criteria of GDM in 2013, which was later adopted in our hospital on 1st July 2015. GDM was diagnosed as either a FG between 6.0 and 6.9 mmol/L and/or a 2hG between 7.8 and 11.0 mmol/L after OGTT (before 1st July 2015), or FG between 5.1 and 6.9 mmol/L, and/or a 2hG between 8.5 and 11.0 mmol/L after OGTT (after 1st July 2015)^2^. DIP was defined as either a FG ≥ 7.0 mmol/L and/or a 2hG ≥ 11.1 mmol/L^2^. Women who had a normal OGTT result before 24 weeks or did not have any risk factors for GDM would receive OGTT at 24–32 weeks of gestations. Early and late onset GDM was defined as a diagnosis before and after 24 weeks of gestation respectively. Women with GDM received advice on lifestyle modification, self-monitoring of blood glucose and dietitian referral.

Pregnancy and neonatal complications were assessed and defined as follows:

- Any complications: experienced at least one of the complications listed below.
- Gestational hypertension: development of newly-onset hypertension (blood pressure persistently ≥ 140/90 mmHg on two occasions at least 4 h apart) during pregnancy after 20 weeks’ gestation, labour or the puerperium in a previously normotensive non-proteinuric women.
- Pre-eclampsia: gestational hypertension with proteinuria (excretion of 300 mg or more per 24 h).
- Preterm birth: birth between 24 + 0 and 36 + 6 weeks of gestation.
- Stillbirth: baby born with no signs of life after 24 + 0 weeks of gestation.
- Shoulder dystocia: vaginal delivery that required additional obstetric manoeuvres to deliver the fetus after the head has delivered and gentle traction has failed.
- Macrosomia: birth weight ≥ 4000 g.
- Large for gestational age fetus (LGA): birth weight > 90th percentile.
- Low birth weight (LBW): birth weight < 2500 g.
- Small for gestational age fetus (SGA): birth weight < 10th percentile.
- Neonatal hypoglycaemia: received glucose treatment or glucose levels of ≤ 1.7 mmol/L in the first 24 h after birth or ≤ 2.5 mmol/L after the first 24 h.
- Respiratory distress syndrome: radiological evidence of respiratory distress syndrome.

Ethical approval was obtained and the need of the informed was waived by the institutional review board of the University of Hong Kong and the Hospital Authority Hong Kong West Cluster due to its retrospective nature. All methods were performed in accordance with the relevant guidelines and regulations.

### Statistical analysis

Continuous variables were reported as median and interquartile range while categorical variables were reported as number and percentage. Maternal FG and 2 hG levels were analysed as both a categorical and a continuous variable in multivariate logistic regression analyses. For associations of maternal glucose levels with pregnancy outcomes as a categorical variable, we divided the glucose levels into 10 categories, ranging from hypoglycaemic levels (glucose < 3.5 mmol/l), pre-gestational diabetic levels (fasting glucose 3.5–3.9, 4.0-4.1, 4.2–4.4, 4.5–4.7 and 4.8-5.0mmol/l, 2 h glucose 3.5–4.5, 4.6–5.3, 5.4–6.1, 6.2–6.9, 7.0-7.7 and 7.8–8.4 mmol/l), gestational diabetic levels (fasting glucose 5.1–5.3, 5.4–5.9 and 6.0-6.9 mmol/l, 2 h glucose 8.5–9.2 and 9.3–11.0 mmol/l) and diabetic levels (fasting glucose ≥ 7 mmol/l, 2 h glucose ≥ 11.1 mmol/l). The category with the lowest rate of pregnancy complications was used as the reference category for calculation of odds ratios. If there were zero recorded cases of the particular complications in the primary reference category, the category with the second lowest rate of pregnancy complications was used as the reference category instead. For analysis of glucose levels as a continuous variable, odds ratios were calculated for per 1 mmol/L increase as well as per 1 standard deviation (SD) increase in FG and 2 hG levels. To assess for curvilinear associations, odds ratios were calculated with squared terms of the standardised glucose levels added to the model. Sensitivity analysis combining category 1 with 2 and category 9 with 10 was conducted to investigate for potential biases that may be induced by having small sample sizes in category 1 and 10. Models were adjusted for maternal age, ethnicity, parity, gestational age at OGTT and baby’s sex. All statistical analyses were performed in SPSS Statistics Software(version 28.0; IBM Corp, NY, USA). *P* < 0.05 was considered statistically significant.

## Results

**Fig. 1:**
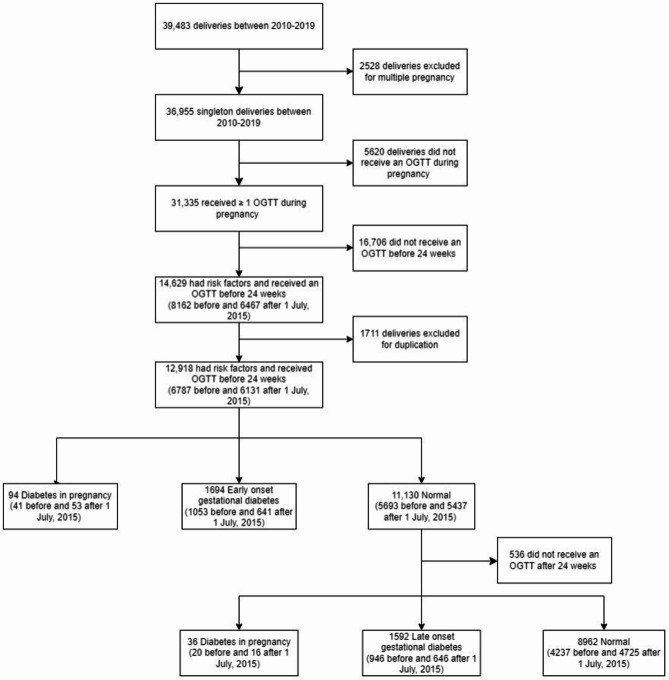
Inclusion and exclusion of the study sample.

There were a total of 39,483 deliveries during the study period. 2528 deliveries were excluded for multiple pregnancy. 5620 deliveries did not receive an OGTT during pregnancy and a further 16,706 deliveries did not have an OGTT done before 24 weeks of pregnancy, leaving 14,629 deliveries that had risk factors and received an early OGTT. After excluding 1711 deliveries due to > 1 pregnant event for the same individual, there were a total of 12,918 women included in the final analysis (Fig. 1). There were no missing data. The overall demographics, pregnancy, and neonatal outcomes of all women that received an early OGTT was shown in Table 1. 84.5% were Chinese and 44.1% were nulliparous. The medians (IQR) of FG and 2 hG were 4.3 mmol/L (4.1–4.6) and 6.1 mmol/L (5.2–7.2) respectively. The median (IQR) gestational age of early OGTT performed was 17.0 weeks (15.4–19.0). The livebirth rate was 99.4%. The commonest pregnancy and neonatal complications were primary Caesarean Sect. (15.4%) and LGA (10.3%) respectively.

**Table 1:** Maternal demographics, pregnancy and neonatal outcomes.

	Total *N* = 12,918
	n (%)/median (IQR)
Maternal age at estimated date of confinement, years	36.0 (33.0–38.0)
Ethnicity	
Chinese	10,912 (84.5)
Others	2006 (15.5)
Nulliparity	5694 (44.1)
Gestational age at early OGTT, weeks	17.0 (15.4–19.0)
Fasting glucose at early OGTT, mmol/L	4.3 (4.1–4.6)
2 h glucose at early OGTT, mmol/L	6.1 (5.2–7.2)
Gestational age at delivery, weeks	39.3 (38.4–40.1)
Pregnancy complications	
Gestational hypertension	332 (2.6)
Pre-eclampsia	248 (1.9)
Preterm birth*	826 (6.4)
Maternal insulin use	118 (0.9)
Primary Caesarean section*	1985 (15.4)
Fetal outcome	
Livebirth	12,837 (99.4)
Miscarriage	28 (0.2)
Stillbirth	34 (0.3)
Neonatal death	19 (0.1)
Baby’s sex*	
Female	6258 (48.5)
Male	6632 (51.5)
Birth weight, gram*	3165.0 (2885.0–3455.0)
Neonatal complications	
Shoulder dystocia^†^	92 (0.7)
Macrosomia^†^	374 (2.9)
Large for gestational age fetus^†^	1325 (10.3)
Low birth weight^†^	937 (7.3)
Small for gestational age fetus^†^	918 (7.1)
Neonatal hypoglycaemia^†^	404 (3.1)
Respiratory distress syndrome^†^	60 (0.5)
NICU admission > 24 h^†^	222 (1.7)

*Miscarriages excluded.

^†^Miscarriages and stillbirths excluded.

### Glucose levels and pregnancy complications

**Fig. 2:**
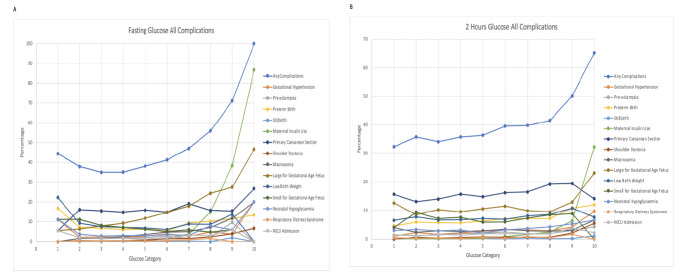
Frequency of pregnancy complications across the categories of maternal fasting and 2 h glucose levels: (**A**) Fasting glucose all complications, (**B**) 2 h glucose all complications.

The number of subjects under each category of FG and 2 hG levels was shown in Table 2. Figure 2 showed the frequency of all complications and Supplemental Figure S1A-O showed the frequency of each complication across the glucose categories. Frequency of developing any complications ranged from 35.0 to 100.0% for FG and 32.3–65.2% for 2hG. Table 3 showed the association of maternal FG and 2 hG levels at early OGTT with each pregnancy outcome.

**Table 2:** The number of women under each category of maternal fasting and 2 h glucose levels.

	1	2	3	4	5	6	7	8	9	10
Fasting Glucose	< 3.5	3.5–3.9	4.0-4.1	4.2–4.4	4.5–4.7	4.8-5.0	5.1–5.3	5.4–5.9	6.0-6.9	≥ 7.0
Number (%)	18 (0.1)	1201 (9.3)	2122 (16.4)	4751 (36.8)	3149 (24.4)	1162 (9.0)	321 (2.5)	127 (1.0)	52 (0.4)	15 (0.1)
2 h Glucose	< 3.5	3.5–4.5	4.6–5.3	5.4–6.1	6.2–6.9	7.0–7.7	7.8–8.4	8.5–9.2	9.3–11.0	≥ 11.1
Number (%)	198 (1.5)	1287 (10.0)	2282 (17.7)	2888 (22.4)	2405 (18.6)	1698 (13.1)	977 (7.6)	615 (4.8)	475 (3.7)	93 (0.7)

**Table 3:** Associations between maternal fasting and 2 h glucose level at early OGTT and pregnancy outcomes.

Glucose category	Fasting glucose			2 h glucose		
	Number (%)	Unadjusted odds ratio (95% confidence intervals)	Adjusted odds ratio (95% confidence intervals)^†^	Number (%)	Unadjusted odds ratio (95% confidence intervals)	Adjusted odds ratio (95% confidence intervals)^†^
Any complications*						
1	8/18 (44.4)	1.484 (0.583–3.777)	1.715 (0.661–4.446)	64/198 (32.3)	1.000	1.000
2	454/1199 (37.9)	1.131 (0.976–1.310)	1.137 (0.978–1.321)	459/1283 (35.8)	1.166 (0.848–1.604)	1.214 (0.877–1.682)
3	741/2116 (35.0)	1.000	1.000	778/2281 (34.1)	1.084 (0.795–1.478)	1.186 (0.864–1.628)
4	1664/4744 (35.1)	1.003 (0.900-1.116)	1.056 (0.946–1.179)	1030/2882 (35.7)	1.164 (0.856–1.584)	1.307 (0.954–1.791)
5	1196/3142 (38.1)	1.140 (1.017–1.279)	1.226 (1.089–1.379)	873/2399 (36.4)	1.198 (0.879–1.632)	1.375 (1.002–1.888)
6	477/1156 (41.3)	1.304 (1.125–1.510)	1.469 (1.262–1.709)	670/1693 (39.6)	1.371 (1.002–1.876)	1.609 (1.167–2.218)
7	151/321 (47.0)	1.648 (1.301–2.088)	1.801 (1.412–2.298)	388/975 (39.8)	1.384 (1.001–1.914)	1.649 (1.182–2.299)
8	71/127 (55.9)	2.353 (1.639–3.377)	2.835 (1.960–4.101)	255/614 (41.5)	1.487 (1.060–2.086)	1.742 (1.232–2.465)
9	37/52 (71.2)	4.577 (2.496–8.395)	4.962 (2.674–9.208)	237/473 (50.1)	2.103 (1.485–2.978)	2.436 (1.704–3.482)
10	15/15 (100.0)	> 100.000	> 100.000	60/92 (65.2)	3.926 (2.329–6.618)	4.884 (2.862–8.337)
Per 1 mmol/L	Not applicable	1.468 (1.337–1.612)	1.614 (1.464–1.780)	Not applicable	1.106 (1.081–1.131)	1.131 (1.105–1.158)
Per 1 SD^§^	Not applicable	1.162 (1.120–1.205)	1.205 (1.160–1.252)	Not applicable	1.172 (1.131–1.215)	1.215 (1.171–1.261)
For glucose squared^¶^	Not applicable	1.088 (1.043–1.135)	1.130 (1.082–1.181)	Not applicable	1.121 (1.078–1.165)	1.170 (1.123–1.218)
Gestational hypertension						
1	2/18 (11.1)	5.641 (1.260–25.250)	6.229 (1.378–28.143)	2/198 (1.0)	1.000	1.000
2	27/1201 (2.2)	1.038 (0.642–1.678)	1.046 (0.646–1.693)	30/1287 (2.3)	2.339 (0.555–9.864)	2.443 (0.578–10.318)
3	46/2122 (2.2)	1.000	1.000	38/2282 (1.7)	1.660 (0.397–6.930)	1.801 (0.430–7.538)
4	107/4751 (2.3)	1.040 (0.733–1.475)	1.073 (0.756–1.524)	63/2888 (2.2)	2.185 (0.531–8.998)	2.422 (0.587-10.000)
5	85/3149 (2.7)	1.252 (0.871-1.800)	1.287 (0.893–1.856)	66/2405 (2.7)	2.765 (0.672–11.375)	3.125 (0.757–12.897)
6	45/1162 (3.9)	1.818 (1.198–2.760)	1.920 (1.260–2.927)	55/1698 (3.2)	3.281 (0.794–13.554)	3.758 (0.906–15.583)
7	9/321 (2.8)	1.302 (0.631–2.686)	1.290 (0.622–2.677)	31/977 (3.2)	3.211 (0.762–13.530)	3.706 (0.876–15.675)
8	6/127 (4.7)	2.238 (0.937–5.343)	2.481 (1.034–5.953)	18/615 (2.9)	2.955 (0.680-12.848)	3.321 (0.761–14.502)
9	2/52 (3.8)	1.805 (0.426–7.644)	1.794 (0.422–7.631)	20/475 (4.2)	4.308 (0.997–18.608)	4.660 (1.074–20.217)
10	3/15 (20.0)	11.283 (3.080-41.335)	13.505 (3.632–50.220)	9/93 (9.7)	10.500 (2.221–49.636)	11.807 (2.480-56.205)
Per 1 mmol/L	Not applicable	1.536 (1.245–1.894)	1.573 (1.270–1.949)	Not applicable	1.189 (1.118–1.266)	1.198 (1.125–1.275)
Per 1 SD^§^	Not applicable	1.182 (1.089–1.283)	1.193 (1.098–1.297)	Not applicable	1.316 (1.192–1.452)	1.330 (1.206–1.468)
For glucose squared^¶^	Not applicable	1.082 (0.958–1.221)	1.087 (0.962–1.230)	Not applicable	1.293 (1.142–1.463)	1.326 (1.171–1.503)
Pre-eclampsia						
1	1/18 (5.6)	3.968 (0.512–30.751)	4.922 (0.623–38.877)	3/198 (1.5)	1.000	1.000
2	23/1201 (1.9)	1.317 (0.764–2.269)	1.309 (0.758–2.259)	16/1287 (1.2)	0.818 (0.236–2.834)	0.828 (0.238–2.877)
3	31/2122 (1.5)	1.000	1.000	33/2282 (1.4)	0.954 (0.290–3.138)	1.017 (0.308–3.360)
4	84/4751 (1.8)	1.214 (0.802–1.839)	1.297 (0.855–1.967)	56/2888 (1.9)	1.285 (0.399–4.143)	1.400 (0.432–4.537)
5	65/3149 (2.1)	1.422 (0.924–2.188)	1.552 (1.004–2.397)	50/2405 (2.1)	1.380 (0.427–4.465)	1.537 (0.472–5.002)
6	31/1162 (2.7)	1.849 (1.118–3.058)	2.140 (1.288–3.557)	42/1698 (2.5)	1.649 (0.506–5.369)	1.866 (0.569–6.118)
7	5/321 (1.6)	1.067 (0.412–2.765)	1.162 (0.446–3.029)	18/977 (1.8)	1.220 (0.356–4.182)	1.398 (0.405–4.826)
8	3/127 (2.4)	1.632 (0.492–5.412)	2.044 (0.612–6.825)	12/615 (2.0)	1.294 (0.361–4.631)	1.439 (0.399–5.189)
9	5/52 (9.6)	7.176 (2.672–19.269)	7.639 (2.808–20.781)	14/475 (2.9)	1.974 (0.561–6.946)	2.130 (0.601–7.552)
10	0/15 (0.0)	NA^‡^	NA^‡^	4/93 (4.3)	2.921 (0.640-13.328)	3.239 (0.702–14.937)
Per 1 mmol/L	Not applicable	1.367 (1.074–1.740)	1.472 (1.158–1.871)	Not applicable	1.132 (1.051–1.219)	1.143 (1.062–1.231)
Per 1 SD^§^	Not applicable	1.130 (1.028–1.241)	1.163 (1.059–1.277)	Not applicable	1.216 (1.082–1.367)	1.236 (1.099–1.389)
For glucose squared^¶^	Not applicable	1.173 (1.029–1.338)	1.219 (1.067–1.391)	Not applicable	1.243 (1.075–1.437)	1.281 (1.106–1.485)
Preterm birth*						
1	3/18 (16.7)	2.780 (0.796–9.716)	2.857 (0.814–10.032)	9/198 (4.5)	1.000	1.000
2	86/1199 (7.2)	1.074 (0.814–1.418)	1.077 (0.815–1.422)	76/1283 (5.9)	1.322 (0.652–2.683)	1.298 (0.639–2.637)
3	142/2116 (6.7)	1.000	1.000	130/2281 (5.7)	1.269 (0.635–2.535)	1.255 (0.627–2.511)
4	286/4744 (6.0)	0.892 (0.724–1.098)	0.909 (0.738–1.120)	163/2882 (5.7)	1.259 (0.633–2.503)	1.219 (0.612–2.428)
5	198/3142 (6.3)	0.935 (0.748–1.168)	0.950 (0.759–1.190)	152/2399 (6.3)	1.421 (0.714–2.828)	1.389 (0.696–2.772)
6	60/1156 (5.2)	0.761 (0.558–1.038)	0.782 (0.572–1.069)	120/1693 (7.1)	1.602 (0.800-3.207)	1.558 (0.776–3.127)
7	30/321 (9.3)	1.433 (0.949–2.165)	1.443 (0.952–2.188)	70/975 (7.2)	1.624 (0.797–3.309)	1.575 (0.771–3.219)
8	13/127 (10.2)	1.585 (0.871–2.884)	1.715 (0.940–3.129)	44/614 (7.2)	1.621 (0.777–3.383)	1.553 (0.742–3.250)
9	6/52 (11.5)	1.813 (0.761–4.318)	1.753 (0.734–4.185)	51/473 (10.8)	2.538 (1.224–5.262)	2.377 (1.143–4.944)
10	2/15 (13.3)	2.139 (0.478–9.570)	2.435 (0.541–10.957)	11/92 (12.0)	2.852 (1.138–7.146)	2.714 (1.079–6.830)
Per 1 mmol/L	Not applicable	1.072 (0.901–1.276)	1.094 (0.918–1.305)	Not applicable	1.109 (1.063–1.157)	1.102 (1.056–1.151)
Per 1 SD^§^	Not applicable	1.027 (0.960–1.100)	1.036 (0.967–1.109)	Not applicable	1.178 (1.101–1.259)	1.167 (1.090–1.249)
For glucose squared^¶^	Not applicable	1.019 (0.944-1.100)	1.027 (0.951–1.110)	Not applicable	1.142 (1.057–1.234)	1.132 (1.047–1.224)
Stillbirth						
1	0/18 (0.0)	NA^‡^	NA^‡^	0/198 (0.0)	NA^‡^	NA^‡^
2	6/1201 (0.5)	5.322 (1.073–26.410)	5.235 (1.054-26.000)	6/1287 (0.5)	1.331 (0.461–3.846)	1.321 (0.456–3.823)
3	2/2122 (0.1)	1.000	1.000	8/2282 (0.4)	1.000	1.000
4	15/4751 (0.3)	3.357 (0.767–14.694)	3.475 (0.793–15.234)	5/2888 (0.2)	0.493 (0.161–1.509)	0.508 (0.166–1.558)
5	7/3149 (0.2)	2.362 (0.490-11.379)	2.532 (0.523–12.260)	8/2405 (0.3)	0.949 (0.355–2.532)	0.974 (0.364–2.605)
6	2/1162 (0.2)	1.828 (0.257–12.991)	2.048 (0.286–14.651)	0/1698 (0.0)	NA^‡^	NA^‡^
7	1/321 (0.3)	3.313 (0.300-36.636)	3.709 (0.332–41.403)	4/977 (0.4)	1.169 (0.351–3.890)	1.232 (0.368–4.121)
8	0/127 (0.0)	NA^‡^	NA^‡^	1/615 (0.2)	0.463 (0.058–3.708)	0.483 (0.060–3.885)
9	1/52 (1.9)	20.784 (1.855-232.914)	23.723 (2.095–268.650)	1/475 (0.2)	0.600 (0.075–4.806)	0.640 (0.079–5.155)
10	0/15 (0.0)	NA^‡^	NA^‡^	1/93 (1.1)	3.090 (0.382–24.962)	3.065 (0.374–25.103)
Per 1 mmol/L	Not applicable	0.971 (0.404–2.330)	1.060 (0.453–2.482)	Not applicable	0.965 (0.777–1.199)	0.977 (0.785–1.216)
Per 1 SD^§^	Not applicable	0.988 (0.702–1.391)	1.023 (0.734–1.426)	Not applicable	0.945 (0.671–1.332)	0.963 (0.681–1.362)
For glucose squared^¶^	Not applicable	0.967 (0.671–1.394)	1.006 (0.696–1.452)	Not applicable	0.873 (0.623–1.221)	0.887 (0.630–1.249)
Maternal insulin use						
1	0/18 (0.0)	NA^‡^	NA^‡^	0/198 (0.0)	NA^‡^	NA^‡^
2	1/1201 (0.1)	0.353 (0.041–3.024)	0.356 (0.042–3.051)	1/1287 (0.1)	0.443 (0.049–3.966)	0.423 (0.047–3.789)
3	5/2122 (0.2)	1.000	1.000	4/2282 (0.2)	1.000	1.000
4	17/4751 (0.4)	1.520 (0.560–4.127)	1.577 (0.581–4.285)	2/2888 (0.1)	0.395 (0.072–2.157)	0.409 (0.075–2.239)
5	9/3149 (0.3)	1.214 (0.406–3.626)	1.267 (0.423–3.794)	6/2405 (0.2)	1.424 (0.401–5.054)	1.501 (0.422–5.331)
6	22/1162 (1.9)	8.171 (3.086–21.634)	8.912 (3.351–23.704)	14/1698 (0.8)	4.735 (1.556–14.409)	5.075 (1.664–15.482)
7	12/321 (3.7)	16.443 (5.753–46.991)	17.210 (5.971–49.606)	16/977 (1.6)	9.482 (3.162–28.435)	10.382 (3.450–31.240)
8	19/127 (15.0)	74.487 (27.295-203.272)	91.533 (33.164-252.627)	15/615 (2.4)	14.237 (4.708–43.055)	15.370 (5.062–46.665)
9	20/52 (38.5)	264.625 (93.505-748.906)	292.990 (102.062-841.088)	30/475 (6.3)	38.393 (13.459-109.518)	40.446 (14.104-115.988)
10	13/15 (86.7)	2752.100 (488.832-15494.190)	4208.370 (716.450-24719.643)	30/93 (32.3)	271.190 (92.753-792.905)	304.907 (103.091-901.807)
Per 1 mmol/L	Not applicable	12.090 (9.165–15.948)	12.821 (9.682–16.976)	Not applicable	2.339 (2.131–2.567)	2.366 (2.151–2.603)
Per 1 SD^§^	Not applicable	2.645 (2.374–2.947)	2.706 (2.425–3.020)	Not applicable	3.836 (3.310–4.445)	3.907 (3.360–4.542)
For glucose squared^¶^	Not applicable	3.627 (3.090–4.258)	3.727 (3.185–4.362)	Not applicable	4.150 (2.898–5.941)	4.219 (2.950–6.033)
Primary caesarean section*						
1	1/18 (5.6)	0.322 (0.043–2.427)	0.393 (0.049–3.127)	31/198 (15.7)	1.000	1.000
2	192/1199 (16.0)	1.043 (0.859–1.267)	1.047 (0.853–1.286)	168/1283 (13.1)	0.812 (0.535–1.231)	0.806 (0.519–1.251)
3	327/2116 (15.5)	1.000	1.000	319/2281 (14.0)	0.876 (0.586–1.308)	0.970 (0.634–1.485)
4	702/4744 (14.8)	0.950 (0.824–1.096)	1.069 (0.919–1.244)	453/2282 (15.7)	1.005 (0.676–1.493)	1.159 (0.761–1.765)
5	499/3142 (15.9)	1.033 (0.887–1.202)	1.213 (1.032–1.427)	355/2399 (14.8)	0.936 (0.627–1.395)	1.118 (0.731–1.708)
6	171/1156 (14.8)	0.950 (0.777–1.161)	1.240 (1.000-1.538)	275/1693 (16.2)	1.045 (0.697–1.566)	1.294 (0.841–1.989)
7	61/321 (19.0)	1.284 (0.948–1.737)	1.594 (1.147–2.217)	161/975 (16.5)	1.066 (0.701–1.620)	1.382 (0.884–2.160)
8	20/127 (15.7)	1.023 (0.625–1.672)	1.583 (0.930–2.694)	118/614 (19.2)	1.282 (0.831–1.975)	1.635 (1.029–2.597)
9	8/52 (15.4)	0.995 (0.464–2.132)	1.028 (0.457–2.311)	92/473 (19.5)	1.301 (0.833–2.032)	1.554 (0.964–2.503)
10	4/15 (26.7)	1.989 (0.630–6.286)	4.100 (1.142–14.721)	13/92 (14.1)	0.886 (0.440–1.786)	1.130 (0.533–2.393)
Per 1 mmol/L	Not applicable	1.060 (0.940–1.195)	1.274 (1.122–1.447)	Not applicable	1.062 (1.031–1.094)	1.099 (1.065–1.135)
Per 1 SD^§^	Not applicable	1.023 (0.976–1.072)	1.099 (1.046–1.155)	Not applicable	1.101 (1.050–1.153)	1.162 (1.104–1.222)
For glucose squared^¶^	Not applicable	1.003 (0.952–1.057)	1.082 (1.022–1.145)	Not applicable	1.105 (1.048–1.165)	1.186 (1.121–1.256)
Shoulder dystocia^#^						
1	0/18 (0.0)	NA^‡^	NA^‡^	0/198 (0.0)	NA^‡^	NA^‡^
2	6/1193 (0.5)	1.182 (0.420–3.330)	1.177 (0.418–3.316)	9/1277 (0.7)	1.606 (0.651–3.963)	1.575 (0.637–3.890)
3	9/2114 (0.4)	1.000	1.000	10/2273 (0.4)	1.000	1.000
4	25/4729 (0.5)	1.243 (0.579–2.668)	1.252 (0.583–2.691)	17/2877 (0.6)	1.345 (0.615–2.943)	1.333 (0.608–2.920)
5	25/3135 (0.8)	1.880 (0.876–4.036)	1.910 (0.886–4.116)	18/2391 (0.8)	1.717 (0.791–3.727)	1.730 (0.796–3.763)
6	16/1154 (1.4)	3.288 (1.449–7.465)	3.277 (1.436–7.476)	12/1693 (0.7)	1.615 (0.696–3.748)	1.632 (0.702–3.798)
7	5/320 (1.6)	3.713 (1.236–11.149)	3.727 (1.229–11.296)	7/971 (0.7)	1.643 (0.624–4.330)	1.638 (0.619–4.330)
8	3/127 (2.4)	5.659 (1.513–21.164)	5.632 (1.498–21.169)	4/613 (0.7)	1.486 (0.465–4.756)	1.472 (0.459–4.727)
9	2/51 (3.9)	9.546 (2.010-45.345)	8.737 (1.834–41.621)	10/472 (2.1)	4.898 (2.027–11.835)	4.685 (1.926–11.400)
10	1/15 (6.7)	16.706 (1.982-140.833)	17.207 (2.015-146.932)	5/91 (5.5)	13.157 (4.402–39.325)	12.958 (4.301–39.041)
Per 1 mmol/L	Not applicable	1.912 (1.397–2.616)	1.941 (1.437–2.622)	Not applicable	1.283 (1.150–1.431)	1.282 (1.148–1.430)
Per 1 SD^§^	Not applicable	1.288 (1.139–1.456)	1.295 (1.152–1.457)	Not applicable	1.483 (1.248–1.764)	1.481 (1.244–1.762)
For glucose squared^¶^	Not applicable	1.615 (1.279–2.040)	1.611 (1.272–2.039)	Not applicable	1.356 (1.081–1.701)	1.361 (1.083–1.709)
Macrosomia^#^						
1	0/18 (0.0)	NA^‡^	NA^‡^	8/198 (4.0)	1.000	1.000
2	19/1193 (1.6)	0.905 (0.518–1.580)	0.923 (0.527–1.615)	31/1277 (2.4)	0.591 (0.268–1.305)	0.695 (0.312–1.547)
3	37/2114 (1.8)	1.000	1.000	66/2273 (2.9)	0.710 (0.336–1.501)	0.880 (0.412–1.876)
4	117/4729 (2.5)	1.424 (0.980–2.068)	1.366 (0.939–1.988)	75/2877 (2.6)	0.636 (0.302–1.337)	0.827 (0.389–1.757)
5	112/3135 (3.6)	2.080 (1.428–3.029)	1.969 (1.347–2.877)	70/2391 (2.9)	0.716 (0.340–1.511)	0.952 (0.447–2.031)
6	55/1154 (4.8)	2.809 (1.840–4.289)	2.488 (1.622–3.815)	58/1693 (3.4)	0.843 (0.396–1.791)	1.154 (0.536–2.484)
7	16/320 (5.0)	2.954 (1.624–5.376)	2.643 (1.441–4.851)	28/971 (2.9)	0.705 (0.317–1.571)	0.947 (0.420–2.135)
8	9/127 (7.1)	4.281 (2.019–9.079)	3.533 (1.650–7.565)	17/612 (2.8)	0.677 (0.288–1.595)	0.917 (0.385–2.184)
9	6/51 (11.8)	7.485 (3.008–18.627)	6.815 (2.697–17.219)	15/472 (3.2)	0.780 (0.325–1.869)	1.036 (0.427–2.515)
10	3/15 (20.0)	14.034 (3.801–51.817)	12.932 (3.386–49.385)	6/91 (6.6)	1.676 (0.564–4.981)	2.222 (0.736–6.711)
Per 1 mmol/L	Not applicable	2.355 (1.938–2.861)	2.203 (1.801–2.695)	Not applicable	1.048 (0.984–1.117)	1.072 (1.006–1.142)
Per 1 SD^§^	Not applicable	1.397 (1.295–1.507)	1.361 (1.258–1.472)	Not applicable	1.077 (0.975–1.191)	1.116 (1.010–1.234)
For glucose squared^¶^	Not applicable	1.459 (1.327–1.605)	1.406 (1.275–1.550)	Not applicable	1.053 (0.943–1.177)	1.108 (0.990–1.241)
Large for gestational age fetus^#^						
1	1/18 (5.6)	0.690 (0.091–5.219)	0.600 (0.078–4.596)	25/198 (12.6)	1.000	1.000
2	75/1193 (6.3)	0.787 (0.594–1.044)	0.791 (0.595–1.052)	109/1277 (8.5)	0.646 (0.406–1.026)	0.719 (0.449–1.151)
3	166/2114 (7.9)	1.000	1.000	233/2273 (10.3)	0.790 (0.509–1.228)	0.898 (0.573–1.407)
4	436/4729 (9.2)	1.192 (0.989–1.436)	1.140 (0.944–1.376)	274/2877 (9.5)	0.728 (0.470–1.128)	0.858 (0.549–1.341)
5	368/3135 (11.7)	1.561 (1.288–1.891)	1.492 (1.227–1.813)	253/2391 (10.6)	0.819 (0.528–1.270)	0.972 (0.621–1.523)
6	170/1154 (14.7)	2.027 (1.615–2.544)	1.839 (1.460–2.317)	195/1693 (11.5)	0.901 (0.577–1.406)	1.094 (0.694–1.724)
7	57/320 (17.8)	2.543 (1.833–3.528)	2.373 (1.698–3.316)	96/971 (9.9)	0.759 (0.475–1.214)	0.899 (0.557–1.452)
8	31/127 (24.4)	3.789 (2.453–5.853)	3.241 (2.080–5.052)	58/613 (9.5)	0.723 (0.439–1.191)	0.867 (0.521–1.443)
9	14/51 (27.5)	4.440 (2.353–8.380)	4.281 (2.235–8.199)	61/472 (12.9)	1.027 (0.624–1.690)	1.245 (0.749–2.072)
10	7/15 (46.7)	10.268 (3.678–28.666)	9.223 (3.219–26.422)	21/91 (23.1)	2.076 (1.091–3.950)	2.456 (1.272–4.742)
Per 1 mmol/L	Not applicable	2.280 (2.001–2.598)	2.157 (1.887–2.466)	Not applicable	1.058 (1.022–1.096)	1.074 (1.036–1.113)
Per 1 SD^§^	Not applicable	1.379 (1.311–1.452)	1.350 (1.281–1.422)	Not applicable	1.094 (1.034–1.156)	1.120 (1.058–1.185)
For glucose squared^¶^	Not applicable	1.414 (1.332–1.501)	1.376 (1.294–1.464)	Not applicable	1.063 (1.000-1.131)	1.098 (1.031–1.170)
Low birth weight^#^						
1	4/18 (22.2)	3.513 (1.143–10.798)	3.980 (1.282–12.360)	13/198 (6.6)	1.000	1.000
2	110/1193 (9.2)	1.249 (0.969–1.610)	1.255 (0.972–1.620)	99/1277 (7.8)	1.196 (0.657–2.176)	1.153 (0.632–2.103)
3	159/2114 (7.5)	1.000	1.000	151/2273 (6.6)	1.013 (0.564–1.820)	0.985 (0.547–1.775)
4	335/4729 (7.1)	0.937 (0.770–1.141)	0.966 (0.793–1.177)	195/2877 (6.8)	1.035 (0.579–1.850)	0.999 (0.557–1.791)
5	213/3135 (6.8)	0.896 (0.724–1.109)	0.922 (0.744–1.144)	172/2391 (7.2)	1.103 (0.616–1.977)	1.071 (0.596–1.927)
6	70/1154 (6.1)	0.794 (0.594–1.062)	0.849 (0.634–1.139)	117/1693 (6.9)	1.056 (0.584–1.911)	1.022 (0.563–1.857)
7	28/320 (8.8)	1.179 (0.775–1.795)	1.219 (0.798–1.863)	79/971 (8.1)	1.260 (0.686–2.314)	1.234 (0.669–2.276)
8	11/127(8.7)	1.166 (0.615–2.209)	1.331 (0.700-2.532)	53/613 (8.6)	1.347 (0.718–2.526)	1.295 (0.687–2.439)
9	7/51 (13.7)	1.956 (0.867–4.414)	2.067 (0.911–4.692)	51/472 (10.8)	1.724 (0.915–3.247)	1.629 (0.861–3.082)
10	0/15 (0.0)	NA^‡^	NA^‡^	7/91 (7.7)	1.186 (0.457–3.080)	1.163 (0.445–3.037)
Per 1 mmol/L	Not applicable	0.847 (0.707–1.015)	0.886 (0.737–1.064)	Not applicable	1.052 (1.009–1.096)	1.050 (1.007–1.094)
Per 1 SD^§^	Not applicable	0.937 (0.873–1.006)	0.954 (0.888–1.025)	Not applicable	1.083 (1.015–1.155)	1.080 (1.011–1.153)
For glucose squared^¶^	Not applicable	0.927 (0.861–0.997)	0.943 (0.875–1.017)	Not applicable	1.057 (0.984–1.136)	1.054 (0.979–1.134)
Small for gestational age fetus^#^						
1	2/18 (11.1)	1.420 (0.324–6.229)	1.738 (0.388–7.785)	8/198 (4.0)	1.000	1.000
2	133/1193 (11.1)	1.426 (1.123–1.810)	1.430 (1.124–1.821)	119/1277 (9.3)	2.441 (1.174–5.075)	2.357 (1.129–4.923)
3	171/2114 (8.1)	1.000	1.000	163/2273 (7.2)	1.835 (0.888–3.789)	1.793 (0.864–3.719)
4	333/4729 (7.0)	0.861 (0.710–1.043)	0.899 (0.740–1.092)	220/2877 (7.6)	1.967 (0.957–4.043)	1.951 (0.945–4.031)
5	194/3135 (6.2)	0.750 (0.605–0.928)	0.785 (0.632–0.975)	141/2391 (5.9)	1.488 (0.719–3.081)	1.470 (0.706–3.058)
6	57/1154 (4.9)	0.590 (0.434–0.804)	0.659 (0.482–0.901)	102/1693 (6.0)	1.523 (0.730–3.176)	1.508 (0.719–3.163)
7	19/320 (5.9)	0.717 (0.440–1.170)	0.765 (0.466–1.254)	71/971 (7.3)	1.874 (0.887–3.957)	1.920 (0.904–4.078)
8	6/127 (4.7)	0.563 (0.245–1.298)	0.670 (0.289–1.552)	52/613 (8.5)	2.201 (1.027–4.718)	2.223 (1.031–4.794)
9	3/51 (5.9)	0.710 (0.219–2.304)	0.807 (0.246–2.643)	42/472 (8.9)	2.320 (1.069–5.036)	2.333 (1.068–5.098)
10	0/15 (0.0)	NA^‡^	NA^‡^	0/91 (0.0)	NA^‡^	NA^‡^
Per 1 mmol/L	Not applicable	0.530 (0.435–0.646)	0.570 (0.465–0.698)	Not applicable	0.973 (0.932–1.016)	0.980 (0.938–1.024)
Per 1 SD^§^	Not applicable	0.781 (0.722–0.843)	0.803 (0.742–0.869)	Not applicable	0.958 (0.895–1.025)	0.968 (0.903–1.038)
For glucose squared^¶^	Not applicable	0.778 (0.720–0.840)	0.799 (0.739–0.865)	Not applicable	0.951 (0.884–1.022)	0.961 (0.892–1.034)
Neonatal hypoglycaemia^#^						
1	2/18 (11.1)	4.354 (0.979–19.368)	4.710 (1.049–21.137)	6/198 (3.0)	1.000	1.000
2	44/1193 (3.7)	1.334 (0.897–1.984)	1.320 (0.886–1.964)	43/1277 (3.4)	1.115 (0.468–2.655)	1.155 (0.484–2.757)
3	59/2114 (2.8)	1.000	1.000	66/2273 (2.9)	0.957 (0.410–2.236)	1.035 (0.442–2.427)
4	144/4729 (3.0)	1.094 (0.804–1.488)	1.143 (0.840–1.556)	91/2877 (3.2)	1.045 (0.452–2.419)	1.143 (0.492–2.657)
5	89/3135 (2.8)	1.018 (0.729–1.421)	1.084 (0.774–1.517)	50/2391 (2.1)	0.683 (0.289–1.614)	0.762 (0.321–1.807)
6	40/1154 (3.5)	1.251 (0.832–1.881)	1.356 (0.899–2.046)	55/1693 (3.2)	1.074 (0.457–2.529)	1.214 (0.513–2.872)
7	10/320 (3.1)	1.124 (0.569–2.220)	1.187 (0.598–2.358)	36/971 (3.7)	1.232 (0.512–2.965)	1.400 (0.579–3.388)
8	10/127 (7.9)	2.977 (1.485–5.969)	3.330 (1.653–6.707)	26/613 (4.2)	1.417 (0.575–3.495)	1.595 (0.643–3.954)
9	3/51 (5.9)	2.177 (0.659–7.190)	2.178 (0.657–7.220)	25/472 (5.3)	1.790 (0.723–4.433)	1.989 (0.799–4.953)
10	3/15 (20.0)	8.708 (2.394–31.676)	10.364 (2.819–38.103)	6/91 (6.6)	2.259 (0.708–7.206)	2.573 (0.801–8.260)
Per 1 mmol/L	Not applicable	1.259 (1.018–1.558)	1.331 (1.077–1.646)	Not applicable	1.101 (1.037–1.168)	1.115 (1.050–1.183)
Per 1 SD^§^	Not applicable	1.094 (1.007–1.189)	1.118 (1.029–1.215)	Not applicable	1.164 (1.059–1.278)	1.187 (1.081–1.305)
For glucose squared^¶^	Not applicable	0.1.078 (0.972–1.195)	1.104 (0.995–1.225)	Not applicable	1.078 (0.971–1.197)	1.109 (0.997–1.233)
Respiratory distress syndrome^#^						
1	0/18 (0.0)	NA^‡^	NA^‡^	1/198 (0.5)	1.000	1.000
2	5/1193 (0.4)	0.984 (0.329–2.944)	0.992 (0.331–2.968)	4/1277 (0.3)	0.619 (0.069–5.567)	0.588 (0.065–5.298)
3	9/2114 (0.4)	1.000	1.000	10/2273 (0.4)	0.871 (0.111–6.835)	0.833 (0.106–6.570)
4	25/4729 (0.5)	1.243 (0.579–2.668)	1.285 (0.597–2.763)	7/2877 (0.2)	0.480 (0.059–3.925)	0.440 (0.054–3.623)
5	10/3135 (0.3)	0.748 (0.304–1.845)	0.771 (0.311–1.910)	14/2391 (0.6)	1.160 (0.152–8.870)	1.082 (0.140–8.335)
6	6/1154 (0.5)	1.222 (0.434–3.443)	(1.294 (0.456–3.669)	5/1693 (0.3)	0.584 (0.068–5.020)	0.536 (0.062–4.656)
7	3/320 (0.9)	2.213 (0.596–8.220)	2.275 (0.605–8.546)	8/971 (0.8)	1.637 (0.204–13.159)	1.493 (0.184–12.129)
8	2/127 (1.6)	3.742 (0.800-17.504)	4.359 (0.925–20.543)	3/613 (0.5)	0.969 (0.100-9.367)	0.857 (0.088–8.374)
9	0/51 (0.0)	NA^‡^	NA^‡^	8/472 (1.7)	3.397 (0.422–27.338)	2.869 (0.352–23.387)
10	0/15 (0.0)	NA^‡^	NA^‡^	0/91 (0.0)	NA^‡^	NA^‡^
Per 1 mmol/L	Not applicable	1.271 (0.768–2.103)	1.308 (0.789–2.169)	Not applicable	1.223 (1.063–1.407)	1.202 (1.044–1.385)
Per 1 SD^§^	Not applicable	1.098 (0.902–1.337)	1.110 (0.912–1.353)	Not applicable	1.374 (1.101–1.716)	1.339 (1.070–1.675)
For glucose squared^¶^	Not applicable	1.176 (0.863–1.602)	1.192 (0.870–1.634)	Not applicable	1.369 (1.022–1.835)	1.334 (0.993–1.792)
NICU admission^#^						
1	1/18 (5.6)	3.494 (0.452–26.987)	3.933 (0.502–30.812)	3/198 (1.5)	1.000	1.000
2	23/1193 (1.9)	1.168 (0.687–1.986)	1.164 (0.684–1.983)	14/1277 (1.1)	0.721 (0.205–2.530)	0.688 (0.196–2.423)
3	35/2114 (1.7)	1.000	1.000	39/2273 (1.7)	1.135 (0.348–3.705)	1.105 (0.337–3.622)
4	82/4729 (1.7)	1.048 (0.703–1.562)	1.095 (0.734–1.635)	40/2877 (1.4)	0.916 (0.281–2.989)	0.859 (0.262–2.816)
5	42/3135 (1.3)	0.807 (0.513–1.268)	0.855 (0.542–1.348)	45/2391 (1.9)	1.247 (0.384–4.049)	1.194 (0.366–3.897)
6	22/1154 (1.9)	1.154 (0.674–1.977)	1.259 (0.732–2.166)	32/1693 (1.9)	1.252 (0.380–4.127)	1.191 (0.359–3.948)
7	10/320 (3.1)	1.916 (0.939–3.908)	2.053 (0.999–4.219)	18/971 (1.9)	1.228 (0.358–4.208)	1.155 (0.335–3.983)
8	4/127 (3.1)	1.932 (0.676–5.522)	2.307 (0.803–6.629)	11/613 (1.8)	1.188 (0.328–4.301)	1.090 (0.299–3.973)
9	3/51 (5.8)	3.712 (1.103–12.491)	3.624 (1.069–12.280)	18/472 (3.8)	2.577 (0.750–8.850)	2.297 (0.664–7.940)
10	0/15 (0.0)	NA^‡^	NA^‡^	2/91 (2.2)	1.461 (0.240–8.896)	1.336 (0.218–8.195)
Per 1 mmol/L	Not applicable	1.283 (0.979–1.681)	1.357 (1.036–1.776)	Not applicable	1.134 (1.049–1.226)	1.120 (1.035–1.211)
Per 1 SD^§^	Not applicable	1.102 (0.992–1.225)	1.126 (1.014–1.251)	Not applicable	1.220 (1.079–1.380)	1.196 (1.056–1.354)
For glucose squared^¶^	Not applicable	1.126 (0.979–1.295)	1.155 (1.003–1.330)	Not applicable	1.229 (1.056–1.429)	1.207 (1.036–1.407)

*Miscarriages were excluded from the analysis.

^†^Adjusted for age, ethnicity, parity, gestational age at oral glucose tolerance test and baby’s sex.

^#^Miscarriages and stillbirths were excluded from the analysis.

^‡^Regression analysis could not be conducted due to zero recorded cases of the complication in the corresponding glucose category.

^§^Odds ratios for an increase in the plasma glucose level by 1 SD (0.39 mmol/L for fasting glucose or 1.58 mmol/L for 2 h glucose).

^¶^Odds ratios with squared terms of standardised glucose values added to the model to explore curvilinear associations.

### Fasting glucose level

The lowest complication rate was observed in category 3 (FG of 4.0-4.1mmol/L) with the frequency of developing any complications being 35.0% (Table 3). A graded positive association was observed between FG levels and pregnancy complications. Categorical analysis found that the aOR for any complications, maternal insulin use, shoulder dystocia, macrosomia, and LGA increased with escalating FG levels. The elevated risk of developing any complications, gestational hypertension, pre-eclampsia, maternal insulin use, primary Caesarean section, shoulder dystocia, macrosomia, and LGA occurred in levels below the current recommended diagnostic cut-off of FG 5.1 mmol/L (category 7). The odds of stillbirth and neonatal intensive care unit (NICU) admission were increased in women with FG ≥ 6.0 mmol/L (category 9).

FG < 4 mmol/L (category 1 and 2) was associated with higher rates of gestational hypertension, stillbirth, LBW, SGA, neonatal hypoglycemia (FG < 3.5 mmol/L for gestational hypertension, LBW and neonatal hypoglycemia; FG 3.5-3.9mmol/L for stillbirth and SGA).

In the sensitivity analysis, category 3 (4.0–4.1 mmol/L) continued to have the lowest complication rate and therefore yielding similar results (Supplemental Table S1 and S2). The frequency of complication across the glucose categories was shown in Supplemental Figure S2A-Q). The trend of increasing aOR with escalating glucose categories was still observed for any complications, maternal insulin use, shoulder dystocia, macrosomia, and LGA. The increased risks of stillbirth, and SGA associated with FG < 4 mmol/L also persisted.

For continuous analysis, there was a 1.205 (1.160–1.252) increase in odds of developing any complications for every 1 SD increase in FG. With both every 1 mmol/L increase and every 1 SD increase in FG, there were also heightened risks of developing gestational hypertension, pre-eclampsia, maternal insulin use, primary Caesarean section, shoulder dystocia, macrosomia, LGA, neonatal hypoglycaemia and NICU admission, but the risk of developing SGA was lowered. Quadratic curvilinear associations between FG levels and pregnancy outcomes were observed in any complications, pre-eclampsia, maternal insulin use, primary Caesarean section, shoulder dystocia, macrosomia, LGA, SGA and NICU admission.

There were no significant changes in the rate of preterm birth and respiratory distress syndrome across the different FG levels in both categorical and continuous analyses.

### Two hours glucose level

The lowest complication rate was observed in category 1 (2hG < 3.5mmol/L) with frequency of 32.3% for developing any complications (Table 3). Categorical analysis revealed increasing aOR for any complications and maternal insulin use with rising 2 hG level, and this increase in risk was observed in levels below the current recommended diagnostic cut-off of 8.5 mmol/L for 2 hG (category 8). Maternal 2 hG ≥ 9.3 mmol/L (category 9) was associated with elevated odds of developing gestational hypertension, preterm birth, shoulder dystocia, and SGA.

In the sensitivity analysis, the glucose category with the lowest complication rate shifted from category 1 to category 3 (2hG of 4.6-5.3mmol/L) (Supplemental Table S1 and S2). The frequency of complication across the glucose categories was shown in Supplemental figure S2A-Q. Increasing aOR with escalating glucose categories were seen for any complications, gestational hypertension, maternal insulin use, and primary Caesarean section.

There was a 1.215 (1.171–1.261) increase in odds of developing any complications for every 1 SD increase in 2 hG level. Elevated risks of developing gestational hypertension, pre-eclampsia, preterm birth, maternal insulin use, primary caesarean section, shoulder dystocia, macrosomia, LGA, LBW, neonatal hypoglycaemia, respiratory distress syndrome, and NICU admission, were also noted with every increase by 1 mmol/L and 1 SD in 2 hG levels. In addition, quadratic curvilinear associations of 2 hG levels with pregnancy complications were observed in any complications, gestational hypertension, pre-eclampsia, preterm birth, maternal insulin use, primary Caesarean section, shoulder dystocia, LGA and NICU admission.

There were no significant changes in the risk of stillbirth in both categorical and continuous analyses.

## Discussion

### Principal findings

This study demonstrated a progressively increased risk of developing hyperglycaemic complications with escalating FG and 2 hG levels among high risk women in a predominately Chinese population. Every glucose unit rise predisposed the pregnant women to a higher risk of adverse perinatal outcomes. If we use the similar approach as International Association of Diabetes and Pregnancy Study Group to define the diagnostic cut-off for late onset GDM, fasting glucose of ≥ 5.1 mmol/L (aOR 1.8 for any complications) and 2 h glucose of ≥ 8.5 mmol/L (aOR 1.74 for any complications) in our cohort roughly corresponded to the consensus of OR 1.75 for developing any complications in an adjusted model. Glucose levels within or close to the hypoglycaemic range may also have additional adverse pregnancy outcomes.

### Comparing existing literature

It remains questionable whether the data from HAPO could directly apply to earlier gestation as FG decreases and insulin resistance increases during pregnancy. In this study, we observed similar findings as the HAPO study, a continuous positive association between glycaemic levels in early pregnancy and pregnancy complications, such as primary Caesarean section, LGA, pre-eclampsia, and shoulder dystocia. Preterm birth was also only significantly related to 2 hG but not to FG. Higher FG was a protective factor for SGA. In contrast, we demonstrated that both FG and 2 hG levels correlated with neonatal hypoglycaemia and NICU admission. Furthermore, we analysed other pregnancy outcomes, and increased risks of maternal insulin use and macrosomia were observed with both FG and 2 hG in our cohort. 2 hG was associated with increased risks of respiratory distress syndrome and LBW but this association was not seen with FG. Abnormal FG or 2hG may represent two distinct pathophysiological conditions, resulting in different pregnancy complications^15^. In addition, there were significant non-linear associations between glucose levels and various complications, which could be due to the attenuated effects of receiving treatment for hyperglycaemia during pregnancy in our cohort.

We demonstrated increased risks of various pregnancy complications at levels below the current diagnostic threshold of early onset GDM recommended by the WHO. Previous studies evaluated the effects of early and late glycaemia on pregnancy outcomes. A meta-analysis which evaluated the relationship between maternal glucose level (below the diagnostic criteria of GDM) and adverse perinatal outcomes in 207,172 women, a positive association was observed across the distribution without a clear threshold. Both FG and 2 hG increased the risk of pregnancy complications, including Caesarean section, induction of labour, LGA, macrosomia, and shoulder dystocia. In addition, FG had a stronger association with these complications than the post-load concentration^8^. Similarly, first trimester FG level below the diagnostic level of early onset GDM or overt diabetes increased the risk of pregnancy complications, and the risk remained even after adjustment for body mass index and excluding women with GDM^16^.

### Clinical and research implications

Screening of early onset GDM is not uncommon. Around one-third of women who had GDM were diagnosed before 24 weeks of gestation among South Asian pregnant women^17^. There was insufficient normative data regarding the association between early pregnancy glycaemia and complications to inform a pregnancy outcome-based diagnostic criteria similar to that of the HAPO study. However, WHO recommended using the same criteria for after 24 weeks of gestation to diagnose early onset GDM. A randomized controlled trial found that detection and treatment of early onset GDM based on the WHO criteria could reduce adverse neonatal outcomes. This was predominated by a reduction in the incidence of neonatal respiratory distress syndrome without altering NICU admission or mortality. 21.8% of women with risk factors were labelled as GDM before 20 weeks of gestation and thus required intensive dietary modification, glucose monitoring, and extra medical care, but one-third of women diagnosed with early onset GDM spontaneously recovered. Women with lower glycaemic levels had similar primary neonatal outcomes but an increased risk of SGA (adjusted RR 1.75) after receiving treatment. In fact, the association of FG < 4mmol/L with increased risk SGA was also observed in our cohort. These provided evidence that the WHO criteria established based on the data of 24–32 gestational weeks from the HAPO study may not be directly applicable for earlier gestation. A non-intervention, observational cohort study aiming to reveal the detrimental effects of glycaemia in early pregnancy would be difficult, given that these women are likely subjected to repeated OGTT at 24–28 weeks of gestation, or received treatment which would alter the natural course. Nonetheless, women had early onset GDM but subsequently a normal OGTT at 24–28 weeks were still at risk of pregnancy complications^18,19^. Women who had a first trimester FG of ≥ 5.1mmol/L, but subsequently a normal OGTT at 24–28 weeks of gestation, still had a residual risk of macrosomia and gestational hypertensive disorders that was similar to women diagnosed with late onset GDM^18^. A higher FG in early gestation also indicated an increased risk of preterm birth and Caesarean section despite a normal OGTT at later gestation^19^. Similarly, early pregnancy haemoglobin A1c (HbA1c) level of 5.7–6.4% (39-46mmol/mol) was associated with an increased risk of adverse pregnancy outcomes even in women without pregestational diabetes mellitus or GDM^20,21^. Therefore, evaluation of glycaemia in early pregnancy could identify women at risk of hyperglycaemic pregnancy complications, irrespective of the subsequent routine glycaemic assessment by OGTT after 24 weeks of gestation. Further research could focus on the prediction of adverse pregnancy outcomes based on glycaemic assessments in early pregnancy, by either FG, 2 hG or HbA1c, or in combination. A modified diagnostic criterion that is specific for the first and second trimester of pregnancy is required and adjustments for ethnicity may also be necessary for global applicability^22^.

### Strengths and limitations

Our study has several strengths. In contrast to the previous studies which mainly used the category with the lowest glucose level or a pre-defined cut-off to investigate the association between pregnancy complications and different glycaemic levels, we used the glucose category with the lowest rate of pregnancy complication as reference to calculate the adjusted odds ratios as lower glucose levels may increase other adverse events, such as LBW and SGA observed in our cohort. Furthermore, glucose categories were defined by the commonly used definitions for hypoglycaemia, GDM and overt diabetes for better clinical correlation. We also examined many clinically relevant outcomes, rather than the commonly used endpoint of later development of GDM in prior studies and gave individual risk estimates. This added valuable information to women and obstetricians who may have distinct perceptions on different complications. Moreover, a large number of subjects in our cohort enabled us to evaluate rarer outcomes.

Our study had limitations. First, the natural history of glycemia in early pregnancy could be altered and thus attenuated the pregnancy outcomes because of several reasons. (1) Women with a normal glucose value could receive treatment due to the other value was above the diagnostic threshold. (2) Women diagnosed with DIP or GDM received dietary modification, glucose monitoring and/or insulin. (3) Physicians were not blinded from the diagnosis which could lead to iatrogenic interventions trying to avert adverse outcomes. Therefore, our results should be interpreted carefully. However, the effect of intervention would likely lead to an underestimation of risk, and our cohort still found an elevated risk of pregnancy complications despite women receiving treatment for hyperglycaemia in pregnancy. In addition, the increase in complication rate was observed in categories below the diagnostic cut-off which encompassed women who were not treated.

Second, this was a single centre study in a predominately Chinese population which could limit the generalizability to other ethnic groups. South Asians could require a lower fasting and post-load glucose threshold to identify women at risk of hyperglyacemia complications than white British women^22^. However, the similar complication rate of primary Caesarean Sect. (14.8–16)^6,16^ and preterm birth (7.1%)^16^ between our cohort and other studies may suggest that our findings could be equally applicable in other settings.

Third, we could not adjust for some important confounding factors, for example gestational weight gain and body mass index, which are known factors that could negatively impact pregnancy outcomes. The interrelated effects of early pregnancy hyperglycaemia, gestational weight gain and body mass index on adverse pregnancy outcomes should be further evaluated. Nonetheless, the overall findings in this study are consistent with the literature and biologically plausible.

Fourth, there may be selection bias as only the high-risk population was selected to undergo early OGTT in our centre and one hour glucose level was not available. The ideal mode of glycaemic assessment and the threshold to define early onset GDM for interventions among both low- and high-risk women during early pregnancy would require further research in an unselected population.

Finally, the frequency of complications in the categories on the extreme ends were small which could induce bias. Nevertheless, we performed sensitivity analysis and yielded similar results.

## Conclusions

Glycaemic levels in early pregnancy among pregnant women with risk factors for gestational diabetes positively associated with pregnancy complications, even at levels below the current recommended diagnostic criteria for GDM. Early glycaemic evaluation is therefore crucial among these women to identify those at risk of hyperglycaemic pregnancy complications.

## Electronic supplementary material

Below is the link to the electronic supplementary material.

Supplementary Material 1

## Data Availability

The datasets generated during and/or analyzed in the current study are available from the corresponding author upon reasonable request. KWC is the guarantor of this work and, as such, had full access to all the data in the study and takes responsibility for the integrity of the data and the accuracy of the data analysis.
