# Supplementary material for: Early pregnancy hyperglycaemia among pregnant women with risk factors for gestational diabetes increases the risk of pregnancy complications
Source: Sci Rep. 2024 Oct 24;14:25157. doi: 10.1038/s41598-024-76497-5 (PMC11502845; doi:10.1038/s41598-024-76497-5)
Supplement: Supplementary file 1 — Supplementary Material 1 [file 41598_2024_76497_MOESM1_ESM.docx]

**Supplementary**

**Supplementary Figure 1A-O. Frequency of pregnancy complications across the categories of maternal fasting and 2 hours glucose levels**

A

B

C

D

E

F

G

H

I

J

K

L

M

N

O

**Supplementary Figure 2A-Q. Frequency of pregnancy complications across the categories of maternal fasting and 2 hours glucose levels in the sensitivity analysis combining category 1 with 2 and category 9 with 10.**

**A**

**
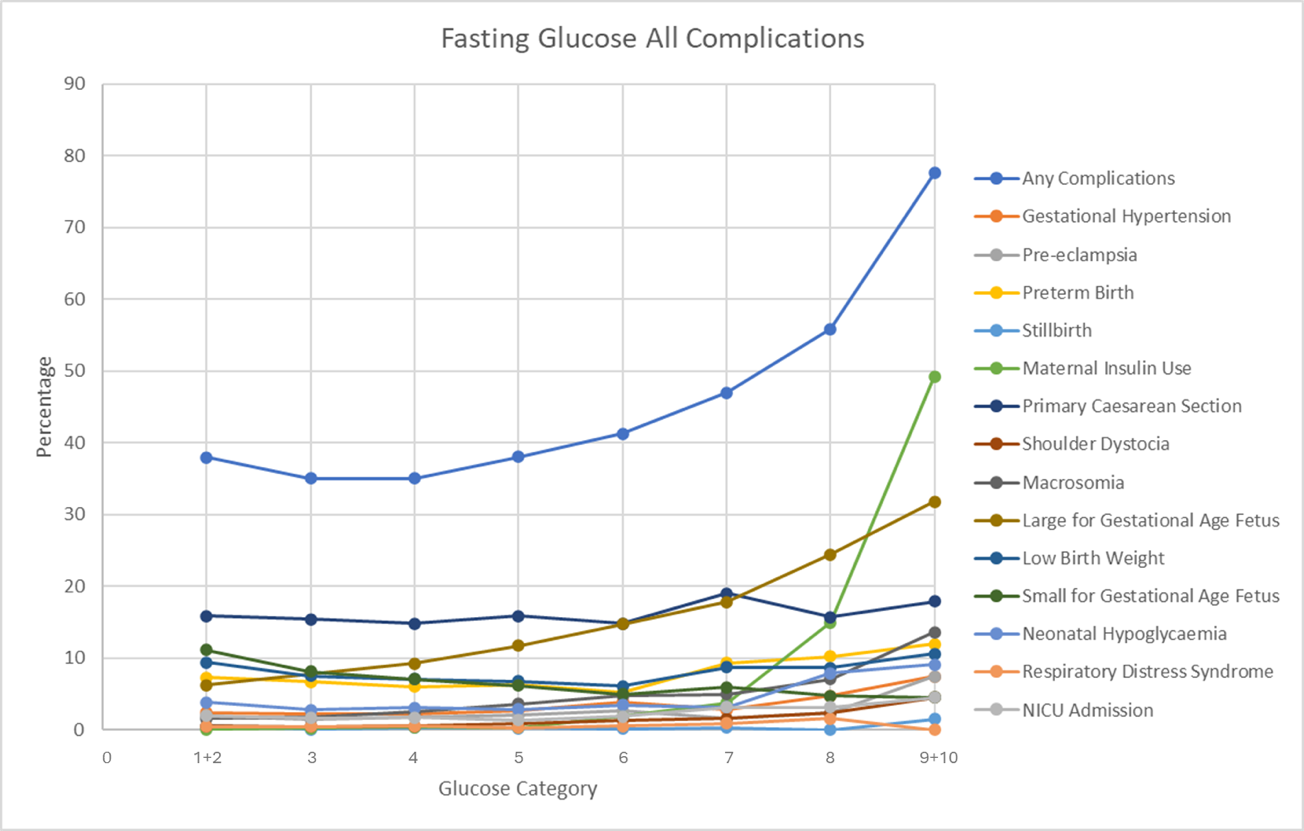
**

**B
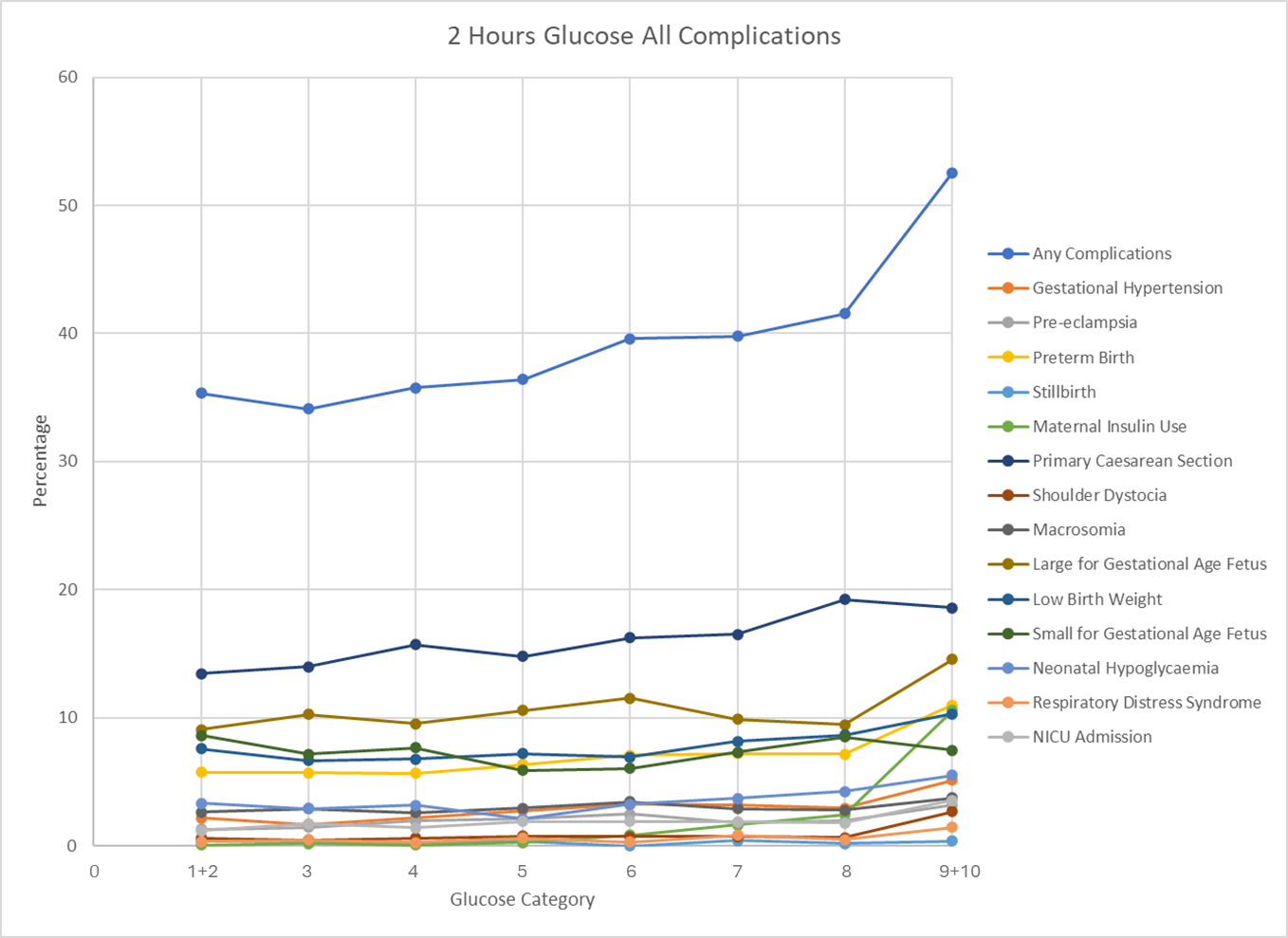
**

**C**

**
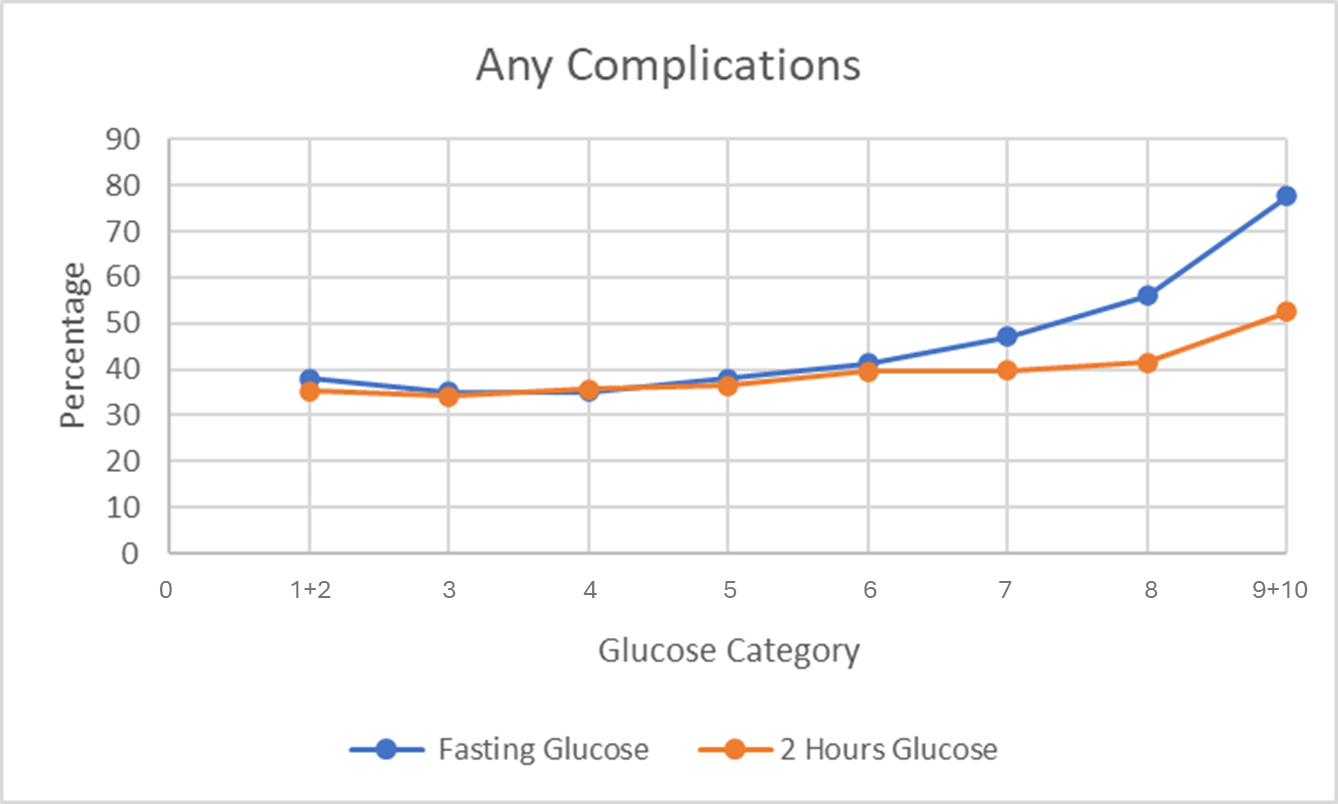
**

**D**

**
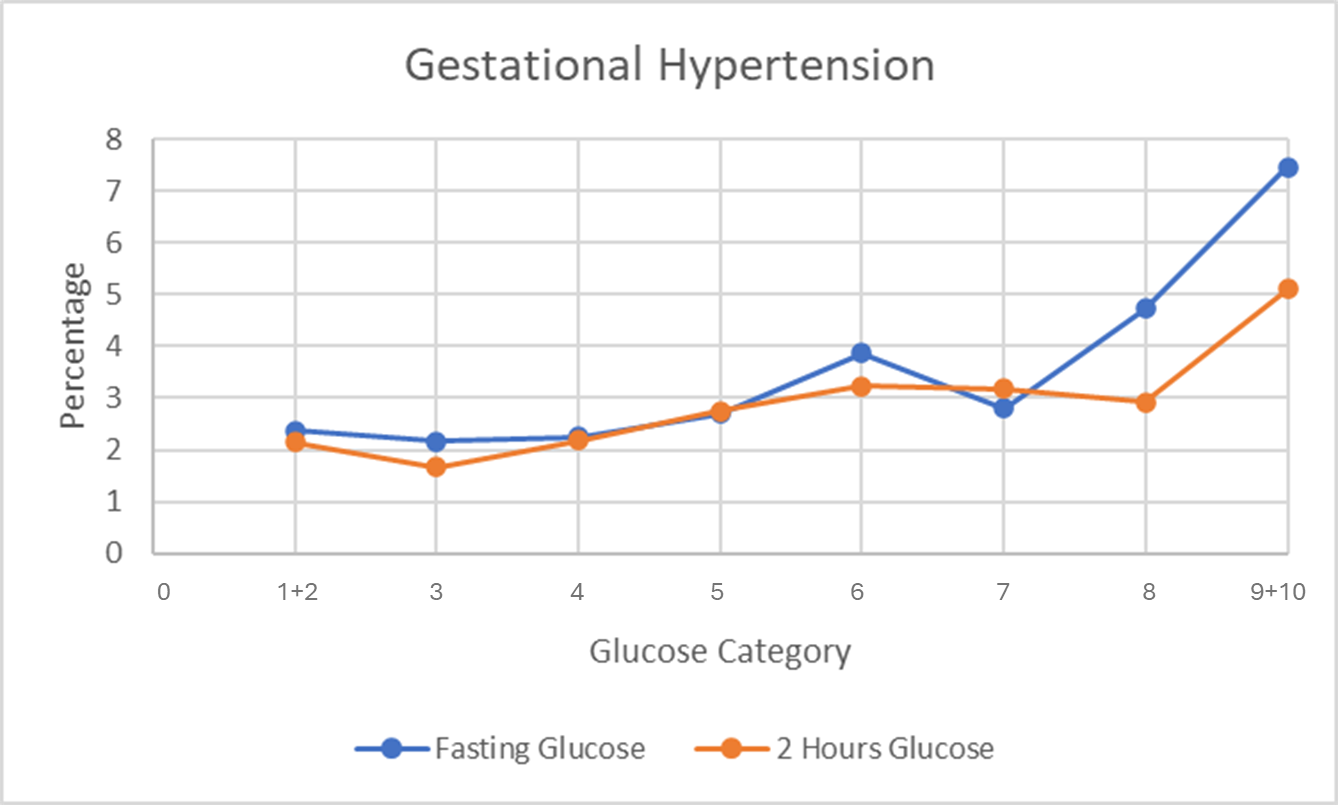
**

**E**

**
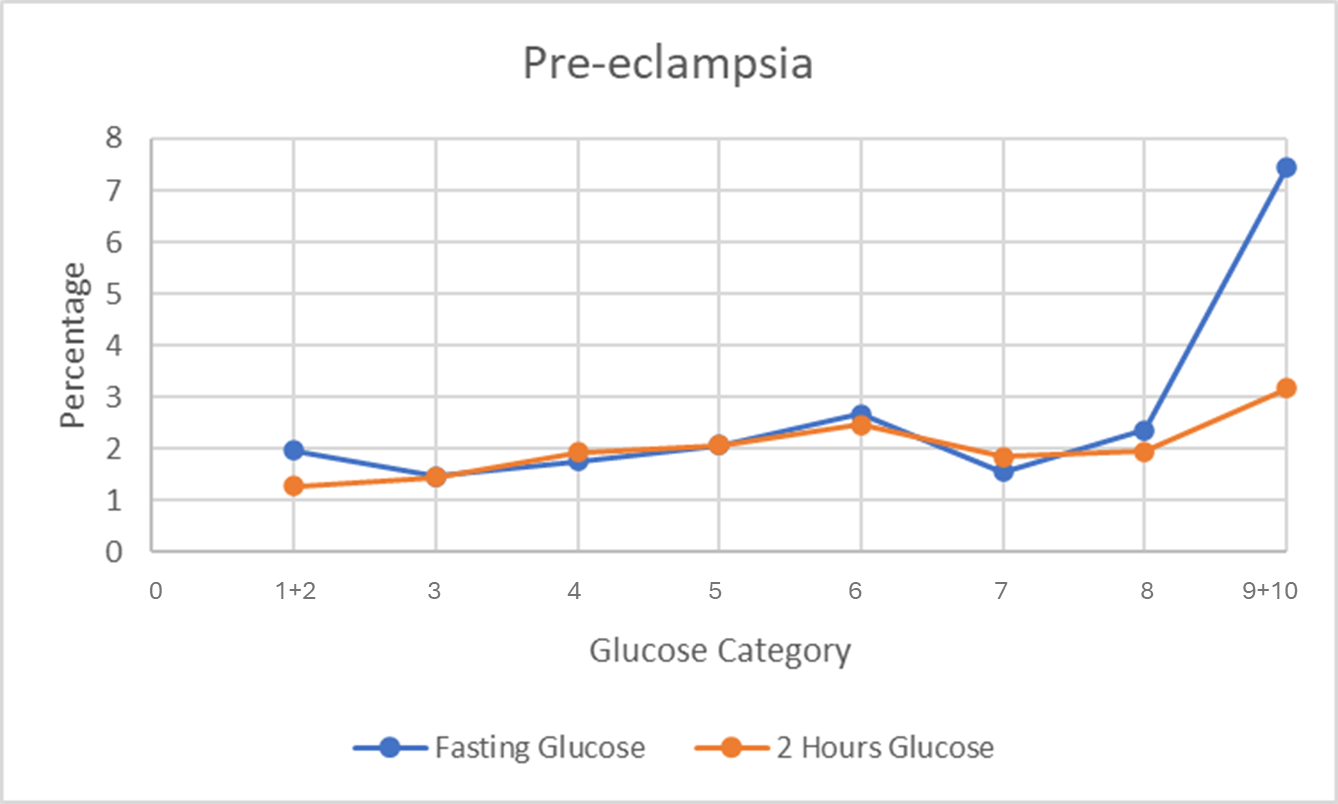
**

**F**

**
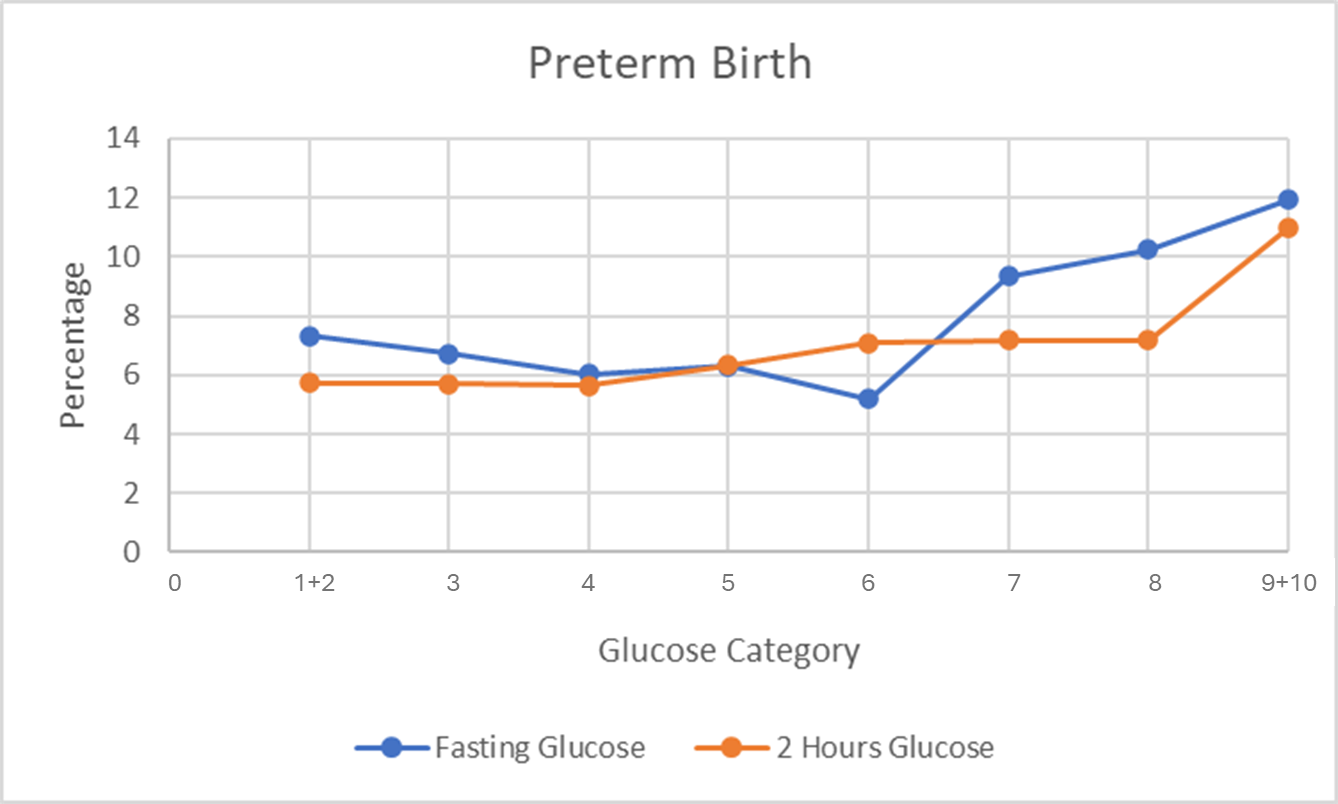
**

**G**

**
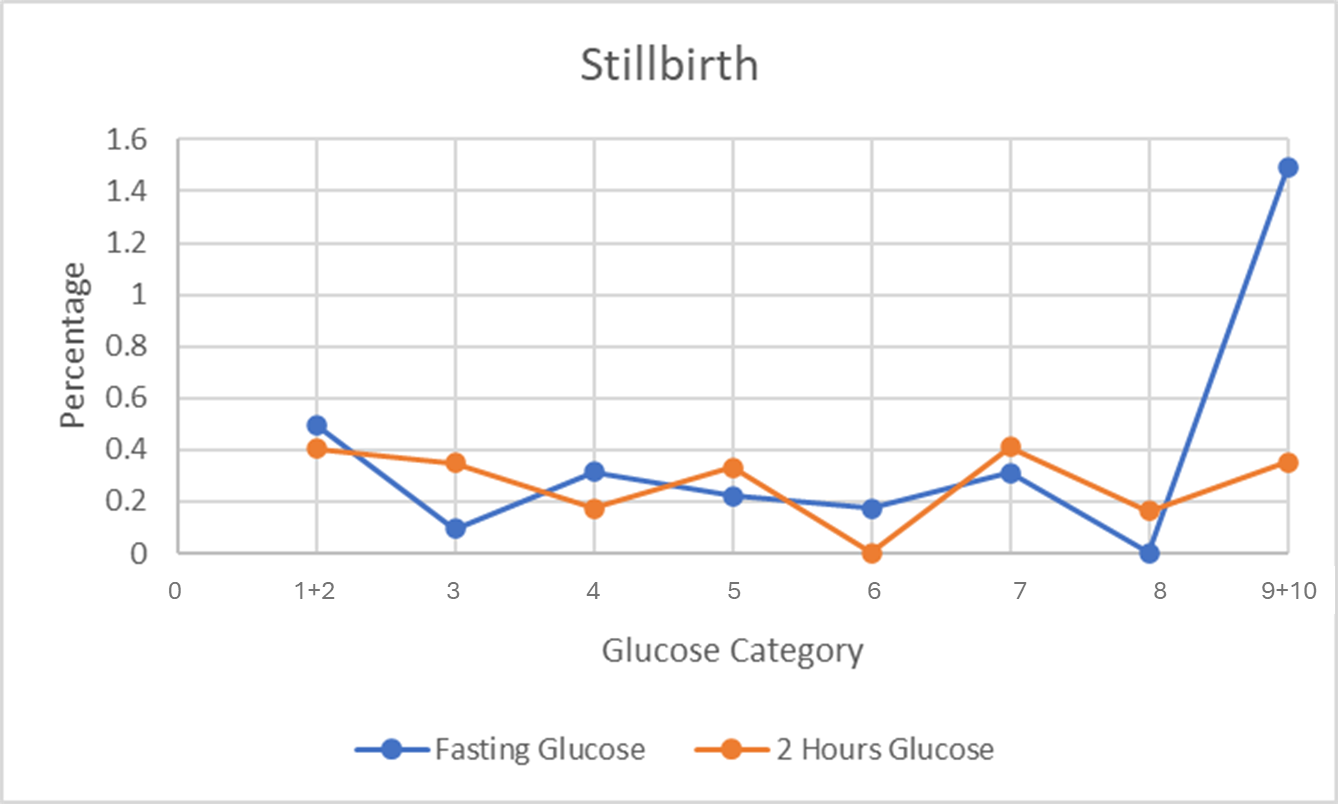
**

**H**

**
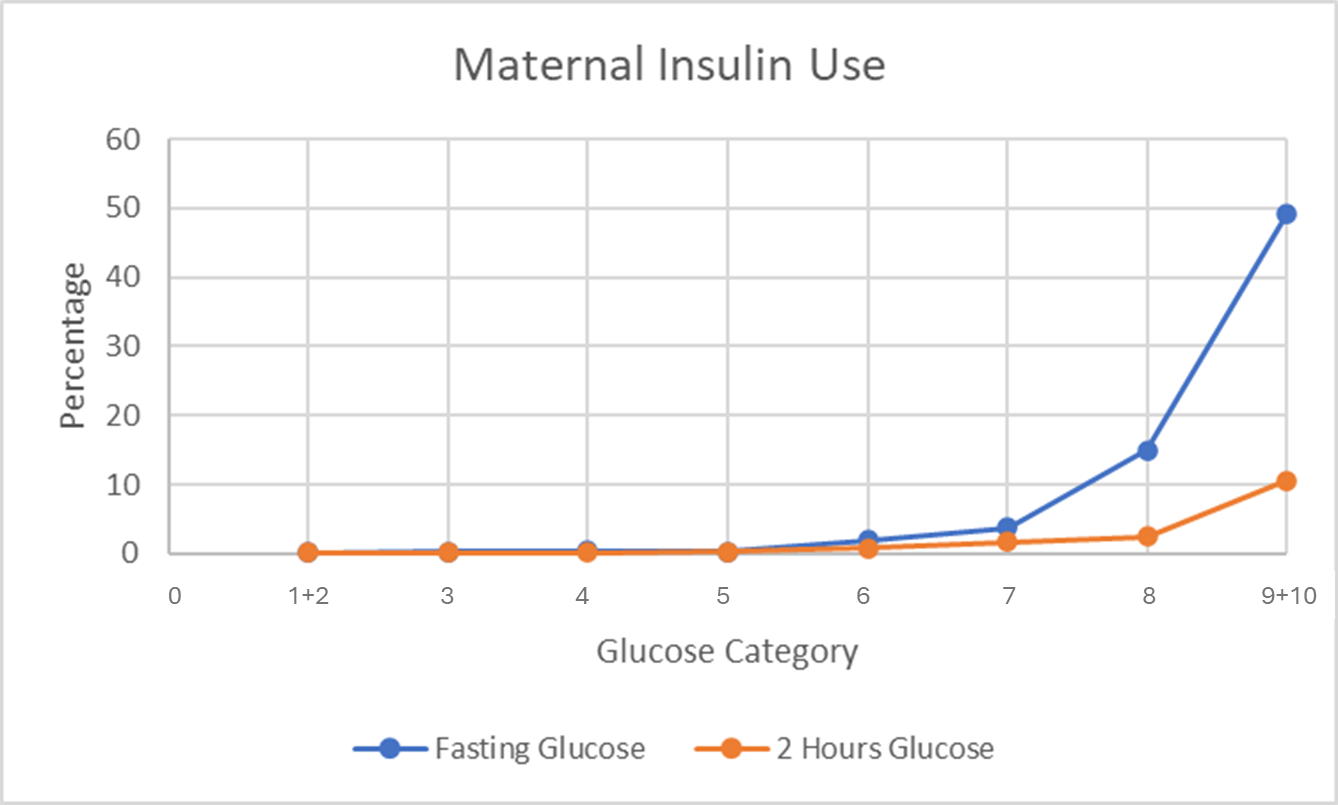
**

**I**

**
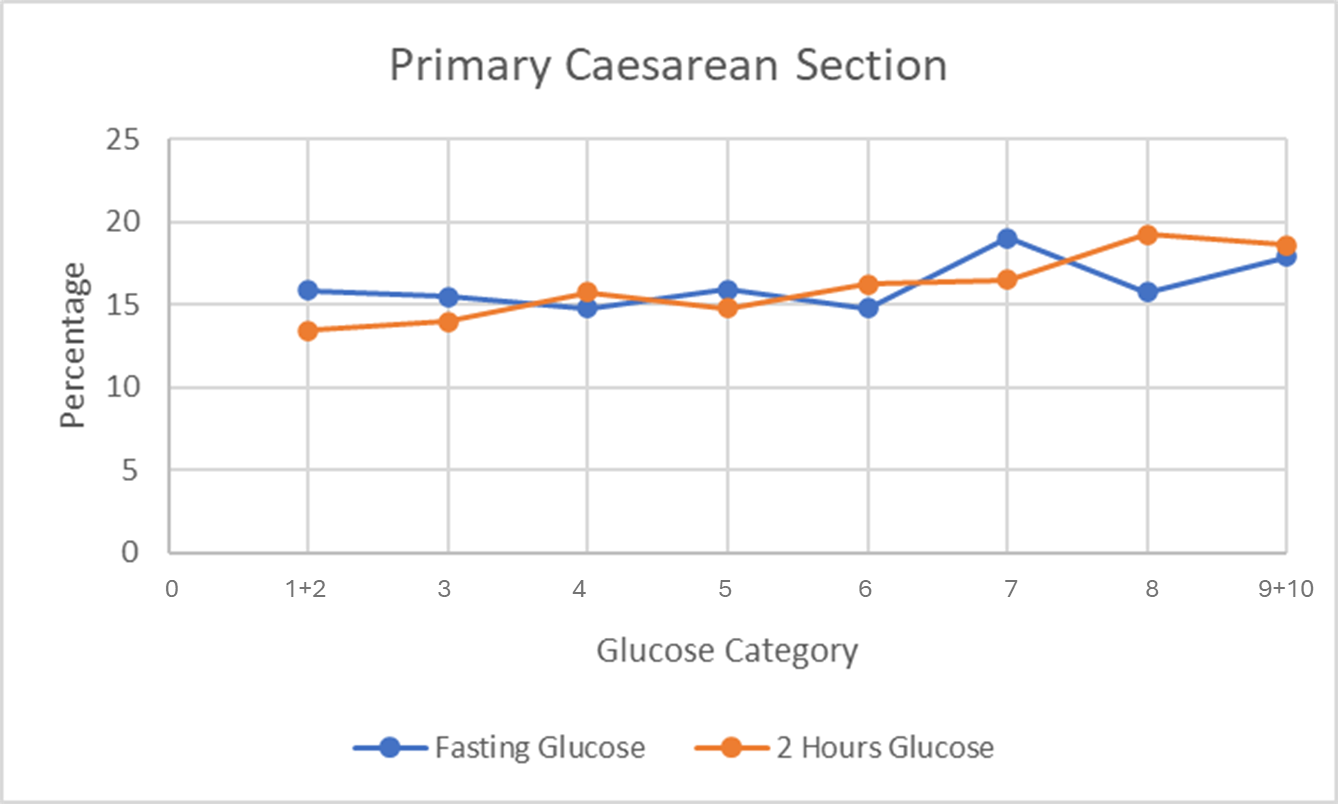
**

**J**

**
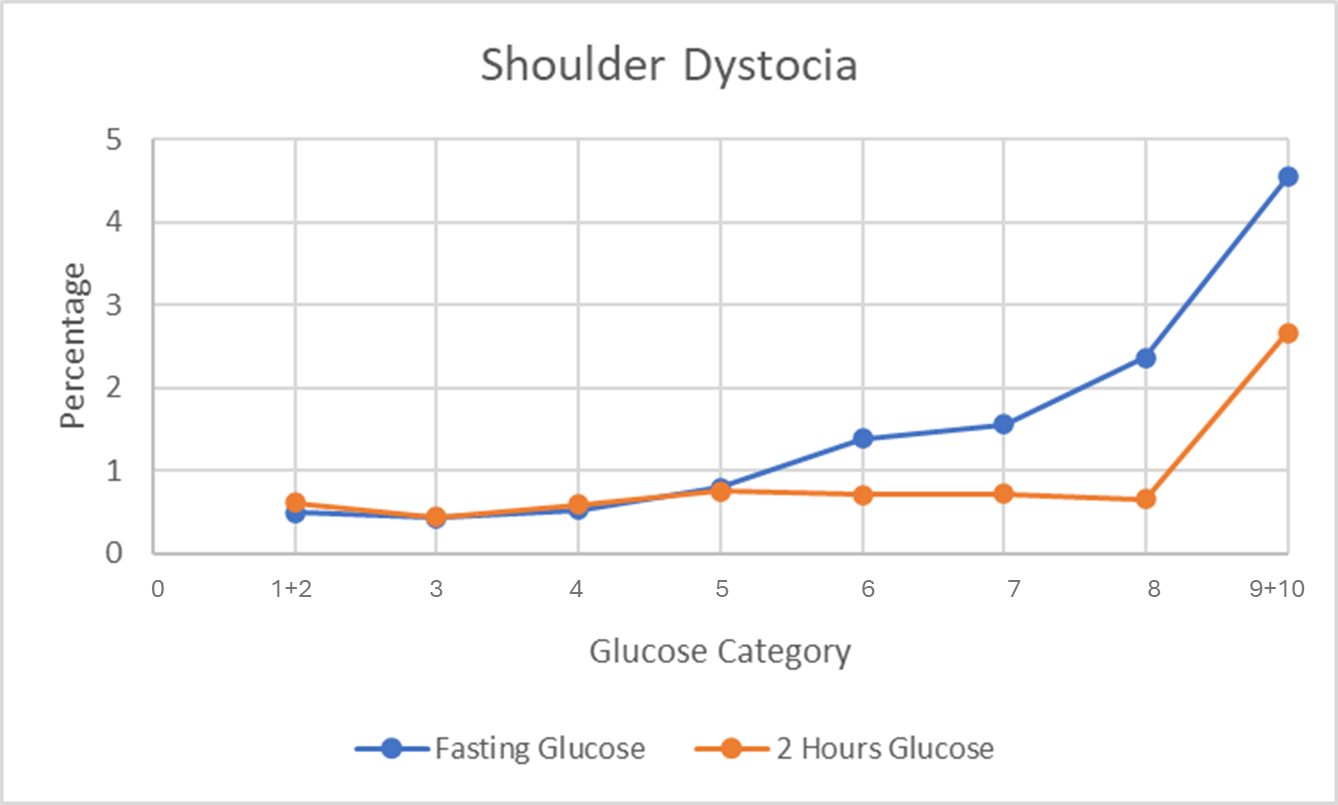
**

**K**

**
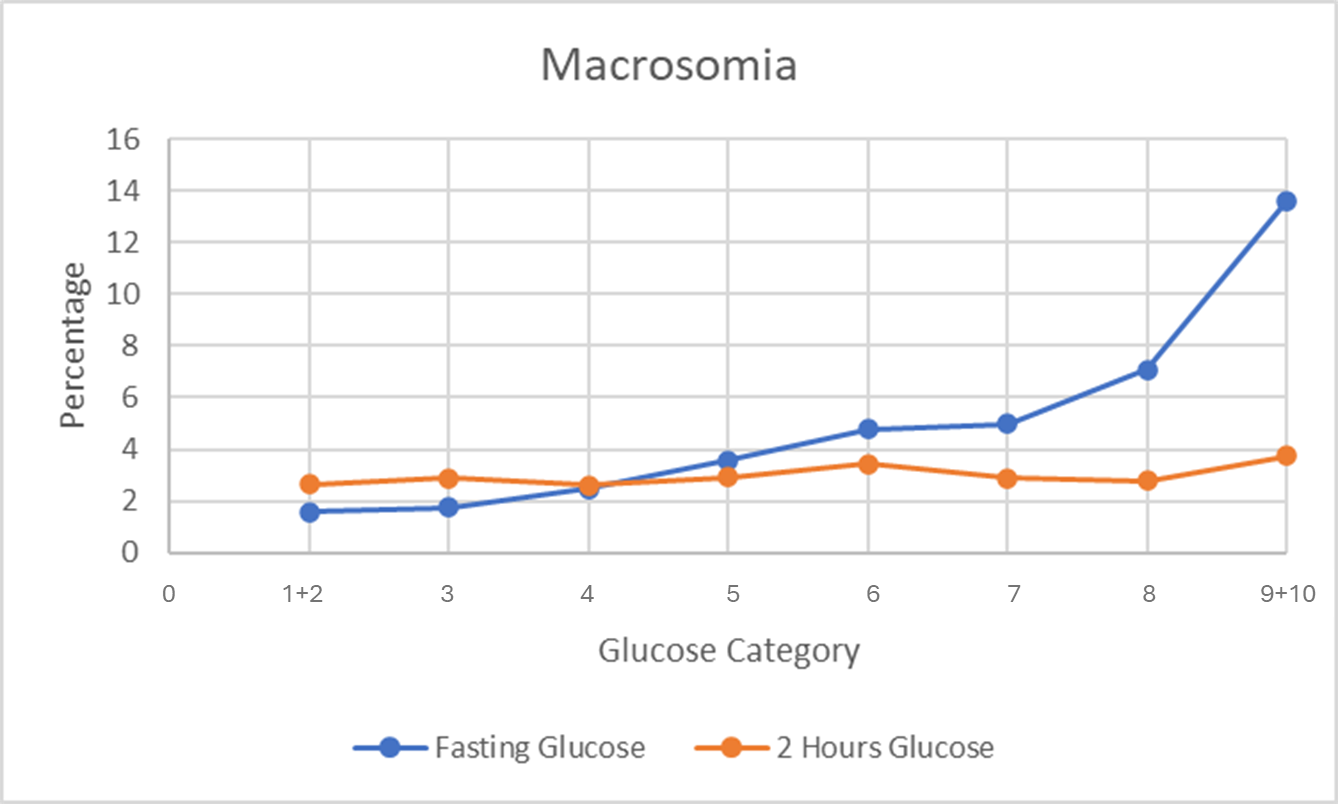
**

**L**

**
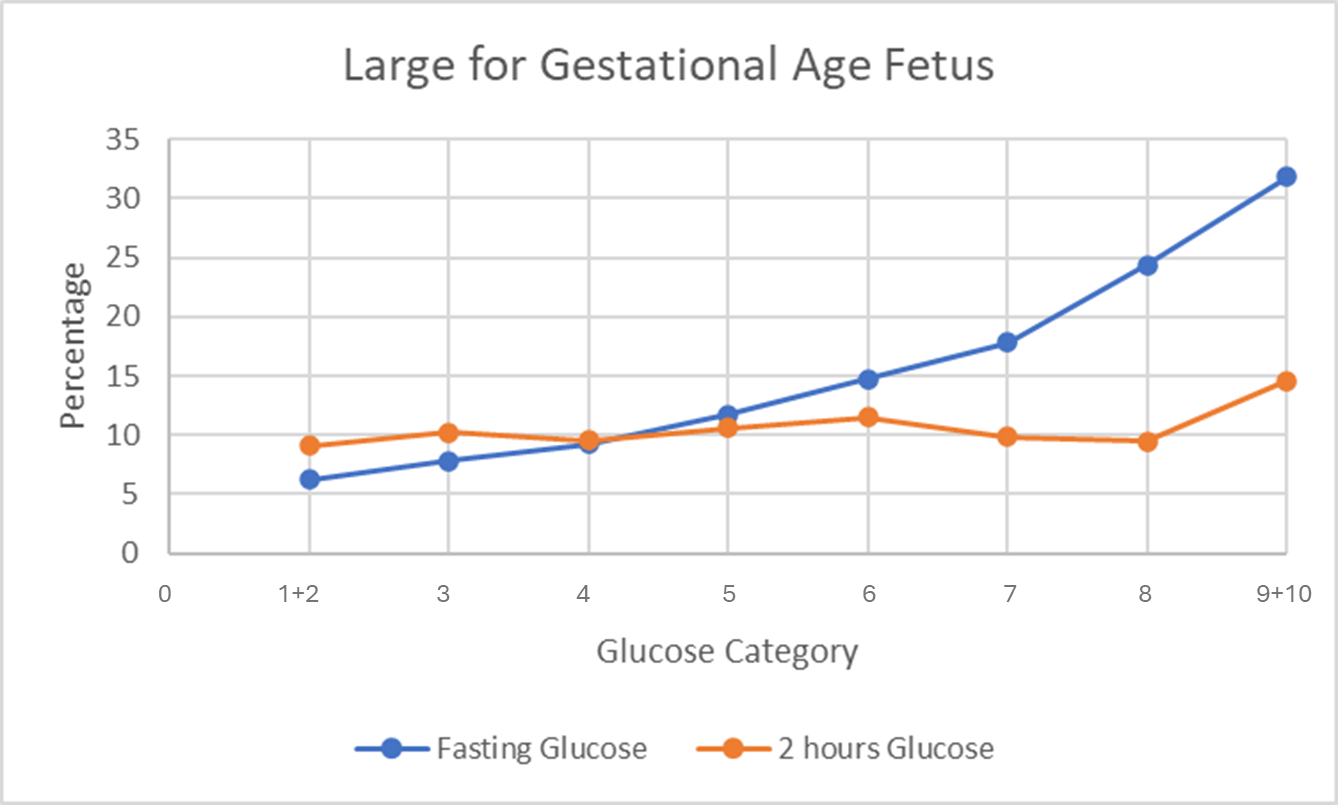
**

**M**

**
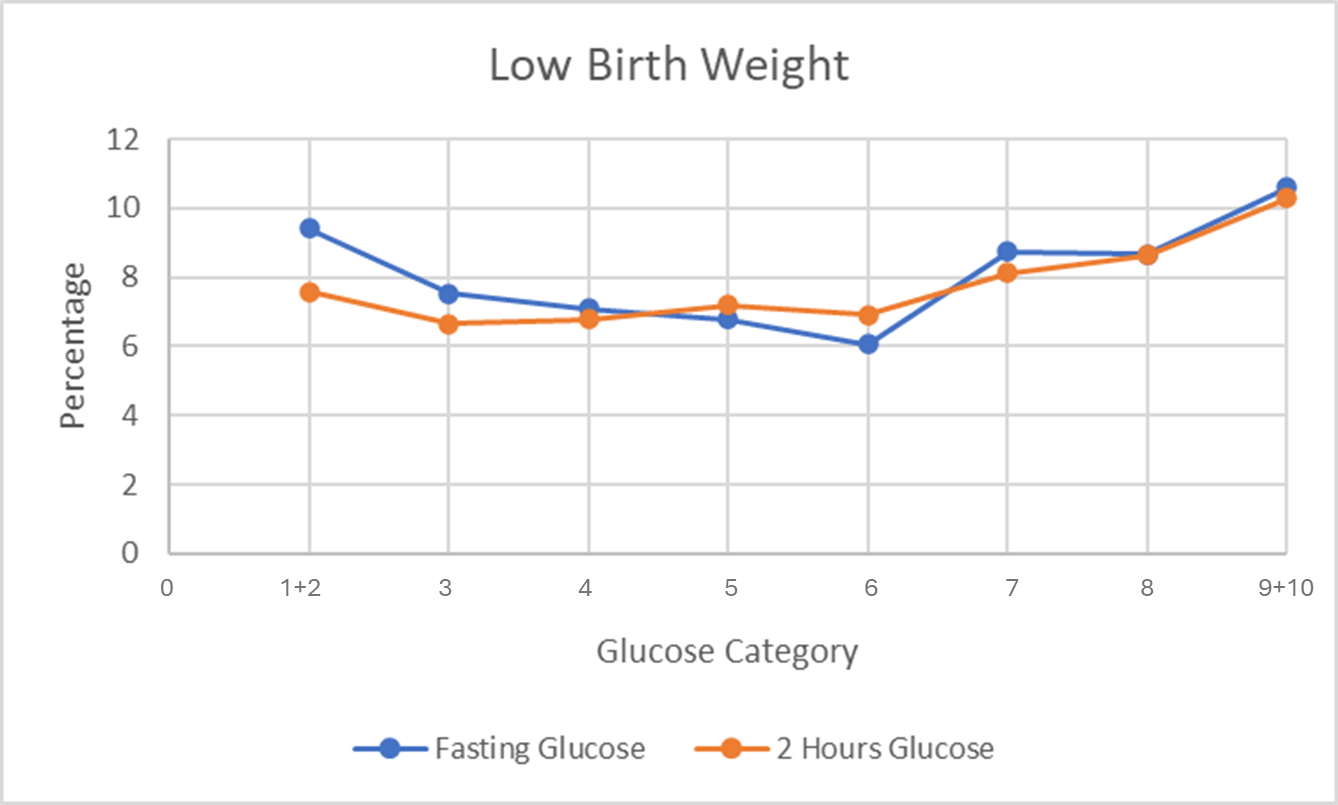
**

**N**

**
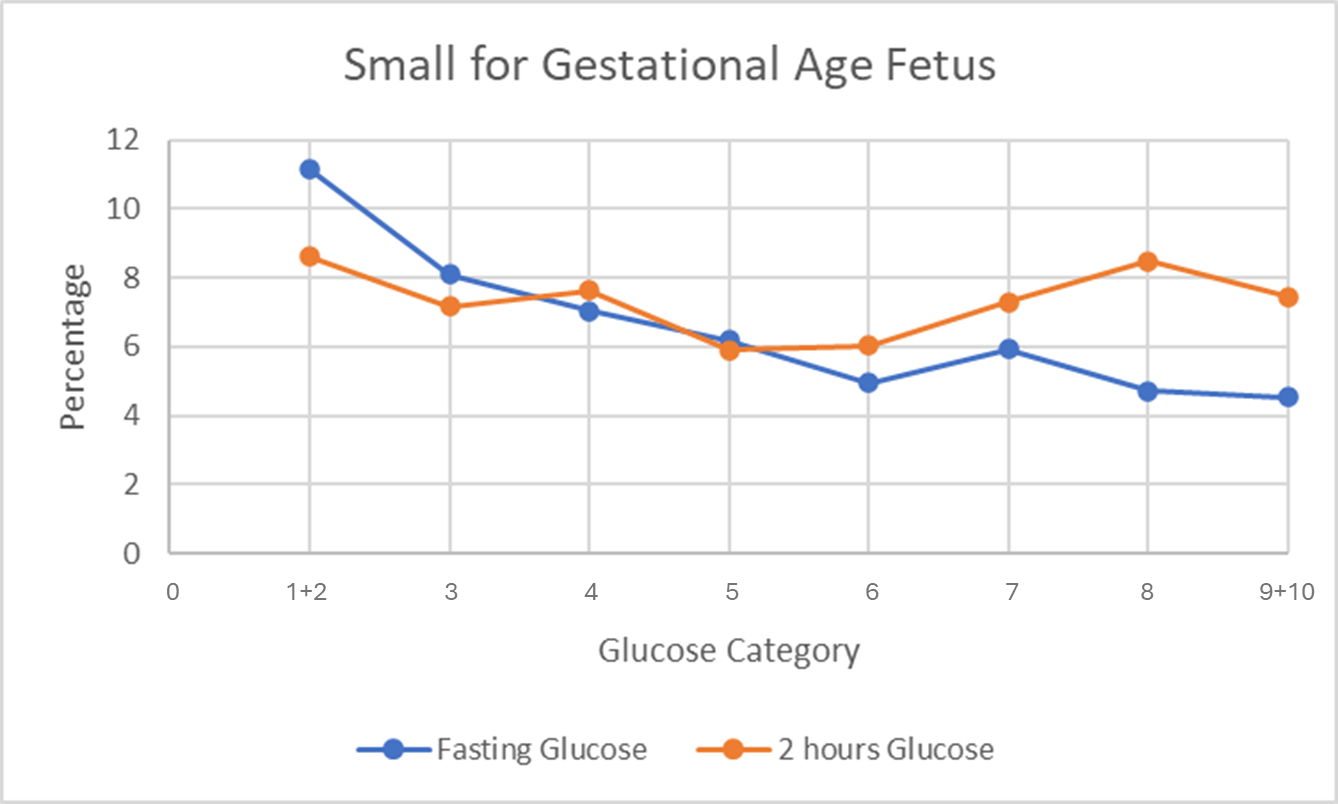
**

**O**

**
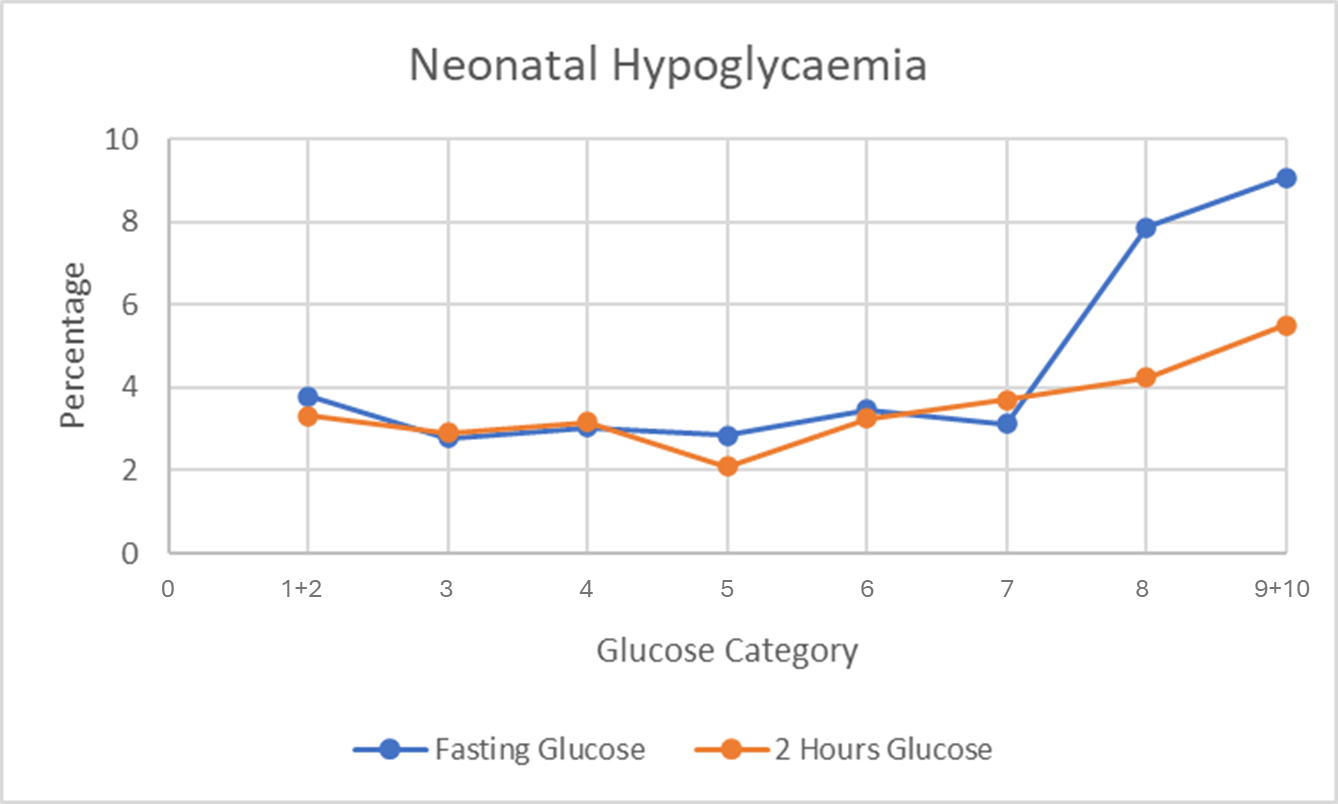
**

**P**

**
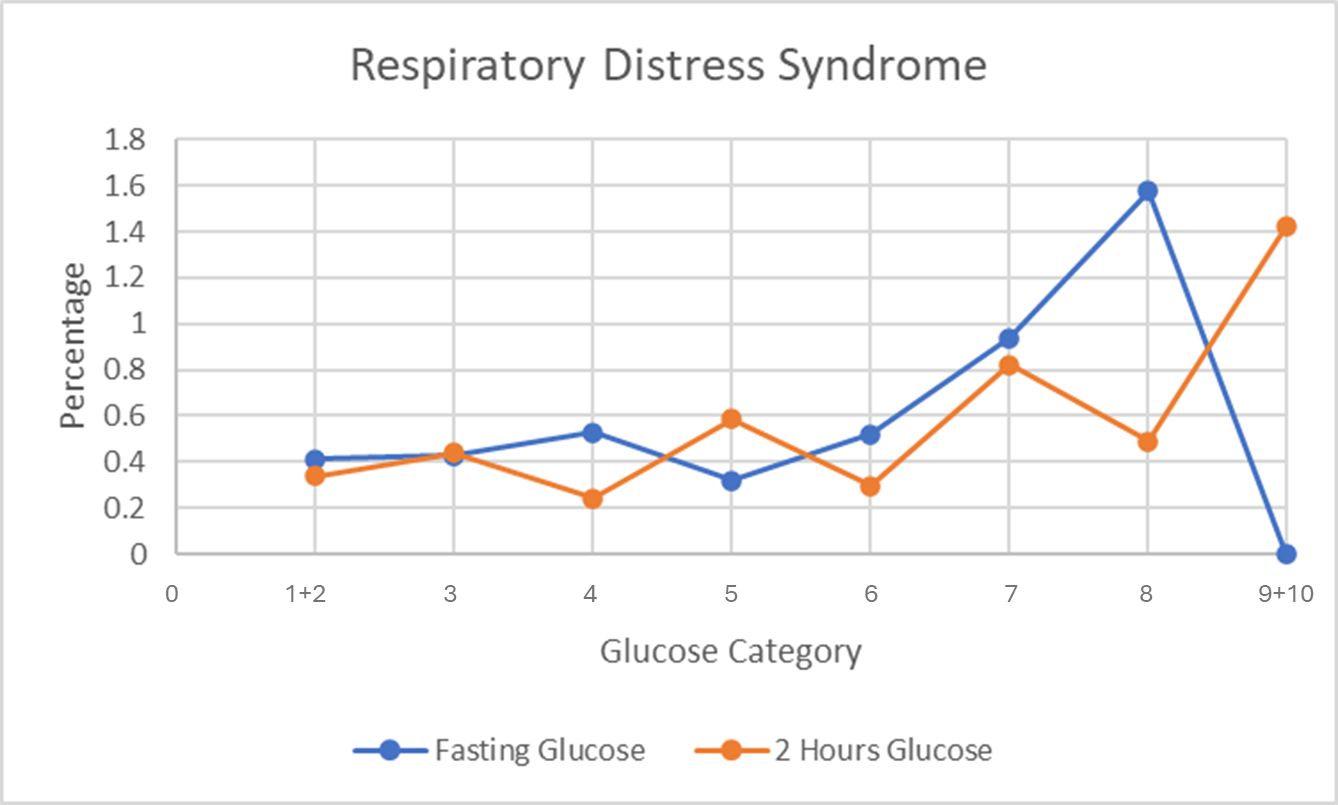
**

**Q**

**
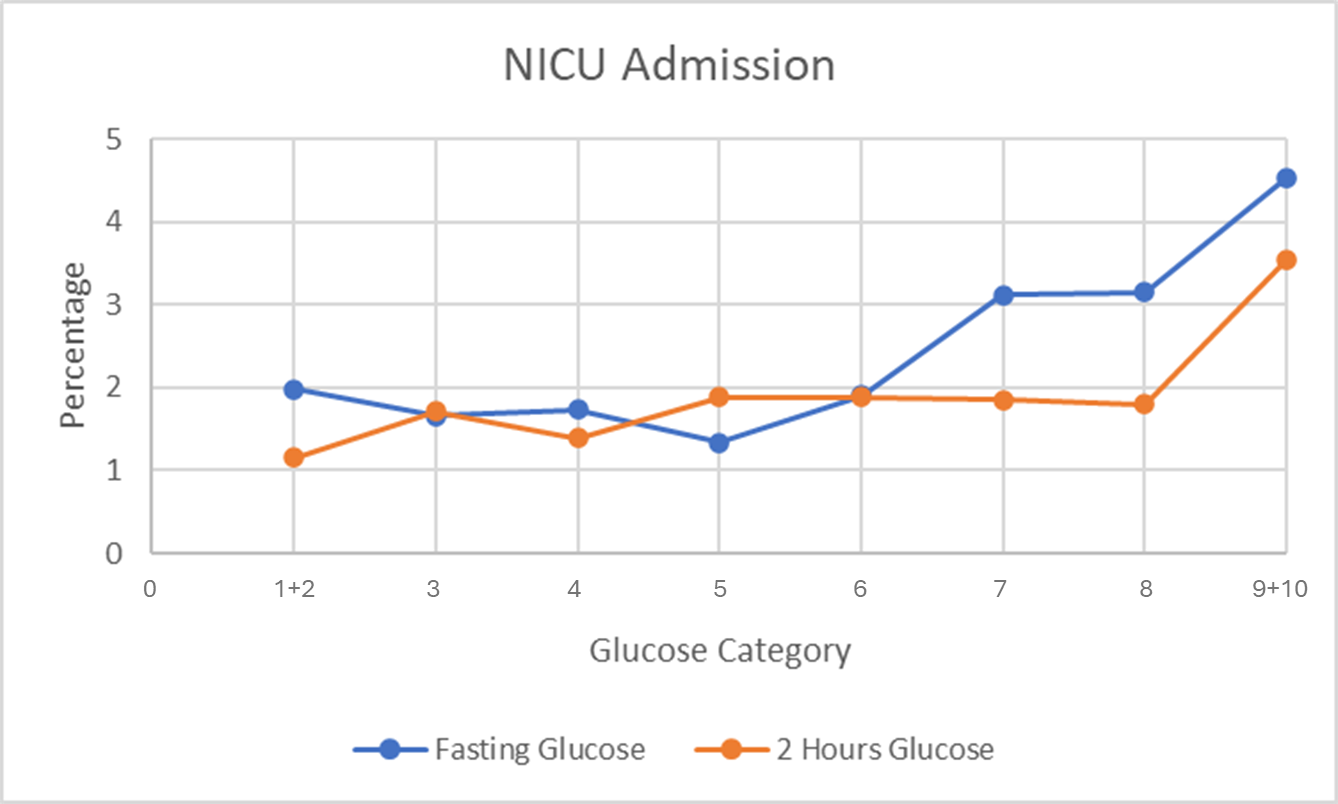
**

| **Supplementary Table 1. Sensitivity analysis combining category 1 with 2 and category 9 with 10 - The number of women under each category of maternal fasting and 2 hours glucose levels.** | | | | | | | | |
| --- | --- | --- | --- | --- | --- | --- | --- | --- |
|  | **1 +2** | **3** | **4** | **5** | **6** | **7** | **8** | **9 + 10** |
| **Fasting Glucose** | < 4.0 | 4.0-4.1 | 4.2-4.4 | 4.5-4.7 | 4.8-5.0 | 5.1-5.3 | 5.4-5.9 | ≥ 6.0 |
| **Number (%)** | 1219 (9.4) | 2122 (16.4) | 4751 (36.8) | 3149 (24.4) | 1162 (9.0) | 321 (2.5) | 127 (1.0) | 67 (0.5) |
| **2 hours Glucose** | < 4.6 | 4.6-5.3 | 5.4-6.1 | 6.2-6.9 | 7.0-7.7 | 7.8-8.4 | 8.5-9.2 | ≥ 9.3 |
| **Number (%)** | 1485 (11.5) | 2282 (17.7) | 2888 (22.4) | 2405 (18.6) | 1698 (13.1) | 977 (7.6) | 615 (4.8) | 568 (4.4) |

| **Supplementary Table 2. Sensitivity analysis combining category 1 with 2 and category 9 with 10 - Associations between Maternal Fasting and 2 Hours Glucose Level at Early OGTT and Pregnancy Outcomes** | | | | | | |
| --- | --- | --- | --- | --- | --- | --- |
| Glucose Category | Fasting Glucose | | | 2 Hours Glucose | | |
|  | Number (%) | Unadjusted Odds Ratio  (95% Confidence Intervals) | Adjusted Odds Ratio  (95% Confidence Intervals)† | Number (%) | Unadjusted Odds Ratio  (95% Confidence Intervals) | Adjusted Odds Ratio  (95% Confidence Intervals)† |
| Any Complications* | | | | | | |
| 1+2 | 462/1217 (38.0) | 1.135 (0.981-1.314) | 1.144 (0.985-1.329) | 523/1481 (35.3) | 1.055 (0.919-1.210) | 0.999 (0.868-1.149) |
| 3 | 741/2116 (35.0) | 1.000 | 1.000 | 778/2281 (34.1) | 1.000 | 1.000 |
| 4 | 1664/4744 (35.1) | 1.003 (0.900-1.116) | 1.056 (0.946-1.179) | 1030/2882 (35.7) | 1.074 (0.957-1.206) | 1.102 (0.979-1.240) |
| 5 | 1196/3142 (38.1) | 1.140 (1.017-1.279) | 1.225 (1.089-1.379) | 873/2399 (36.4) | 1.105 (0.980-1.246) | 1.160 (1.025-1.311) |
| 6 | 477/1156 (41.3) | 1.304 (1.125-1.510) | 1.468 (1.261-1.709) | 670/1693 (39.6) | 1.265 (1.111-1.441) | 1.356 (1.186-1.550) |
| 7 | 151/321 (47.0) | 1.648 (1.301-2.088) | 1.800 (1.411-2.297) | 388/975 (39.8) | 1.277 (1.094-1.491) | 1.390 (1.186-1.629) |
| 8 | 71/127 (55.9) | 2.353 (1.639-3.377) | 2.834 (1.959-4.099) | 255/614 (41.5) | 1.372 (1.143-1.647) | 1.469 (1.218-1.772) |
| 9+10 | 52/67 (77.6) | 6.433 (3.597-11.504) | 7.353 (4.075-13.266) | 297/565 (52.6) | 2.141 (1.777-2.580) | 2.293 (1.892-2.778) |
| Gestational Hypertension | | | | | | |
| 1+2 | 29/1219 (2.4) | 1.100 (0.687-1.760) | 1.111 (0.693-1.779) | 32/1485 (2.2) | 1.301 (0.809-2.091) | 1.245 (0.774-2.004) |
| 3 | 46/2122 (2.2) | 1.000 | 1.000 | 38/2282 (1.7) | 1.000 | 1.000 |
| 4 | 107/4751 (2.3) | 1.040 (0.733-1.475) | 1.072 (0.755-1.522) | 63/2888 (2.2) | 1.317 (0.877-1.977) | 1.344 (0.894-2.020) |
| 5 | 85/3149 (2.7) | 1.252 (0.871-1.800) | 1.285 (0.891-1.853) | 66/2405 (2.7) | 1.666 (1.113-2.494) | 1.734 (1.157-2.599) |
| 6 | 45/1162 (3.9) | 1.818 (1.198-2.760) | 1.916 (1.257-2.920) | 55/1698 (3.2) | 1.977 (1.301-3.004) | 2.084 (1.369-3.174) |
| 7 | 9/321 (2.8) | 1.302 (0.631-2.686) | 1.287 (0.620-2.670) | 31/977 (3.2) | 1.935 (1.197-3.129) | 2.055 (1.268-3.330) |
| 8 | 6/127 (4.7) | 2.238 (0.937-5.343) | 2.473 (1.031-5.931) | 18/615 (2.9) | 1.780 (1.009-3.142) | 1.842 (1.041-3.259) |
| 9+10 | 5/67 (7.5) | 3.640 (1.398-9.476) | 3.786 (1.447-9.904) | 29/568 (5.1) | 3.177 (1.942-5.198) | 3.183 (1.938-5.227) |
| Pre-eclampsia | | | | | | |
| 1+2 | 24/1219 (2.0) | 1.355 (0.791-2.319) | 1.351 (0.788-2.316) | 19/1485 (1.3) | 0.883 (0.500-1.559) | 0.837 (0.473-1.480) |
| 3 | 31/2122 (1.5) | 1.000 | 1.000 | 33/2282 (1.4) | 1.000 | 1.000 |
| 4 | 84/4751 (1.8) | 1.214 (0.802-1.839) | 1.297 (0.855-1.968) | 56/2888 (1.9) | 1.348 (0.873-2.079) | 1.376 (0.891-2.127) |
| 5 | 65/3149 (2.1) | 1.422 (0.924-2.188) | 1.552 (1.005-2.397) | 50/2405 (2.1) | 1.447 (0.929-2.254) | 1.511 (0.968-2.359) |
| 6 | 31/1162 (2.7) | 1.849 (1.118-3.058) | 2.140 (1.288-3.557) | 42/1698 (2.5) | 1.728 (1.091-2.739) | 1.835 (1.155-2.917) |
| 7 | 5/321 (1.6) | 1.067 (0.412-2.765) | 1.162 (0.446-3.029) | 18/977 (1.8) | 1.279 (0.717-2.283) | 1.375 (0.768-2.462) |
| 8 | 3/127 (2.4) | 1.632 (0.492-5.412) | 2.045 (0.613-6.827) | 12/615 (2.0) | 1.356 (0.696-2.642) | 1.415 (0.724-2.767) |
| 9+10 | 5/67 (7.5) | 5.440 (2.046-14.461) | 6.046 (2.250-16.245) | 18/568 (3.2) | 2.230 (1.247-3.991) | 2.266 (1.260-4.076) |
| Preterm Birth* | | | | | | |
| 1+2 | 89/1217 (7.3) | 1.097 (0.833-1.444) | 1.100 (0.835-1.449) | 85/1481 (5.7) | 1.007 (0.760-1.335) | 1.003 (0.756-1.330) |
| 3 | 142/2116 (6.7) | 1.000 | 1.000 | 130/2281 (5.7) | 1.000 | 1.000 |
| 4 | 286/4744 (6.0) | 0.892 (0.724-1.098) | 0.909 (0.737-1.120) | 163/2882 (5.7) | 0.992 (0.782-1.258) | 0.971 (0.765-1.232) |
| 5 | 198/3142 (6.3) | 0.935 (0.748-1.168) | 0.950 (0.759-1.190) | 152/2399 (6.3) | 1.119 (0.879-1.425) | 1.107 (0.868-1.410) |
| 6 | 60/1156 (5.2) | 0.761 (0.558-1.038) | 0.781 (0.571-1.068) | 120/1693 (7.1) | 1.262 (0.977-1.631) | 1.241 (0.959-1.606) |
| 7 | 30/321 (9.3) | 1.433 (0.949-2.165) | 1.443 (0.951-2.187) | 70/975 (7.2) | 1.280 (0.947-1.729) | 1.255 (0.928-1.698) |
| 8 | 13/127 (10.2) | 1.585 (0.871-2.884) | 1.714 (0.940-3.127) | 44/614 (7.2) | 1.277 (0.896-1.820) | 1.237 (0.867-1.765) |
| 9+10 | 8/67 (11.9) | 1.885 (0.883-4.022) | 1.891 (0.884-4.044) | 62/565 (11.0) | 2.039 (1.484-2.802) | 1.937 (1.407-2.667) |
| Stillbirth | | | | | | |
| 1+2 | 6/1219 (0.5) | 5.243 (1.057-26.018) | 5.175 (1.042-25.700) | 6/1485 (0.4) | 1.153 (0.399-3.330) | 1.150 (0.397-3.331) |
| 3 | 2/2122 (0.1) | 1.000 | 1.000 | 8/2282 (0.4) | 1.000 | 1.000 |
| 4 | 15/4751 (0.3) | 3.357 (0.767-14.694) | 3.478 (0.793-15.247) | 5/2888 (0.2) | 0.493 (0.161-1.509) | 0.508 (0.166-1.557) |
| 5 | 7/3149 (0.2) | 2.362 (0.490-11.379) | 2.536 (0.524-12.278) | 8/2405 (0.3) | 0.949 (0.355-2.532) | 0.973 (0.364-2.602) |
| 6 | 2/1162 (0.2) | 1.828 (0.257-12.991) | 2.052 (0.287-14.677) | 0/1698 (0.0) | NA^‡^ | NA^‡^ |
| 7 | 1/321 (0.3) | 3.312 (0.300-36.636) | 3.716 (0.333-41.481) | 4/977 (0.4) | 1.169 (0.351-3.890) | 1.229 (0.368-4.112) |
| 8 | 0/127 (0.0) | NA^‡^ | NA^‡^ | 1/615 (0.2) | 0.463 (0.058-3.708) | 0.482 (0.060-3.876) |
| 9+10 | 1/67 (1.5) | 16.061 (1.438-179.342) | 18.147 (1.616-203.783) | 2/568 (0.4) | 1.004 (0.213-4.743) | 1.057 (0.222-5.027) |
| Maternal Insulin Use | | | | | | |
| 1+2 | 1/1219 (0.1) | 0.348 (0.041-2.979) | 0.352 (0.041-3.013) | 1/1485 (0.1) | 0.384 (0.043-3.437) | 0.366 (0.041-3.275) |
| 3 | 5/2122 (0.2) | 1.000 | 1.000 | 4/2282 (0.2) | 1.000 | 1.000 |
| 4 | 17/4751 (0.4) | 1.520 (0.560-4.127) | 1.568 (0.577-4.261) | 2/2888 (0.1) | 0.395 (0.072-2.157) | 0.408 (0.075-2.231) |
| 5 | 9/3149 (0.3) | 1.214 (0.406-3.626) | 1.256 (0.419-3.763) | 6/2405 (0.2) | 1.424 (0.401-5.054) | 1.494 (0.421-5.306) |
| 6 | 22/1162 (1.9) | 8.171 (3.086-21.634) | 8.813 (3.314-23.436) | 14/1698 (0.8) | 4.735 (1.556-14.409) | 5.050 (1.656-15.402) |
| 7 | 12/321 (3.7) | 16.443 (5.753-46.991) | 16.990 (5.896-48.960) | 16/977 (1.6) | 9.482 (3.162-28.435) | 10.271 (3.414-30.898) |
| 8 | 19/127 (15.0) | 74.487 (27.295-203.272) | 89.445 (32.440-246.623) | 15/615 (2.4) | 14.237 (4.708-43.055) | 15.217 (5.014-46.184) |
| 9+10 | 33/67 (49.3) | 410.947 (151.217-1116.790) | 479.791 (174.182-1321.601) | 60/568 (10.6) | 67.264 (24.336-185.917) | 70.928 (25.529-197.065) |
| Primary Caesarean Section* | | | | | | |
| 1+2 | 193/1217 (15.9) | 1.031 (0.849-1.252) | 1.038 (0.846-1.274) | 199/1481 (13.4) | 0.955 (0.789-1.155) | 0.856 (0.701-1.047) |
| 3 | 327/2116 (15.5) | 1.000 | 1.000 | 319/2281 (14.0) | 1.000 | 1.000 |
| 4 | 702/4744 (14.8) | 0.950 (0.824-1.096) | 1.069 (0.919-1.244) | 453/2282 (15.7) | 1.147 (0.982-1.340) | 1.194 (1.013-1.408) |
| 5 | 499/3142 (15.9) | 1.033 (0.887-1.202) | 1.213 (1.031-1.427) | 355/2399 (14.8) | 1.068 (0.907-1.258) | 1.152 (0.968-1.370) |
| 6 | 171/1156 (14.8) | 0.950 (0.777-1.161) | 1.240 (1.000-1.538) | 275/1693 (16.2) | 1.193 (1.001-1.421) | 1.333 (1.106-1.608) |
| 7 | 61/321 (19.0) | 1.284 (0.948-1.737) | 1.594 (1.146-2.216) | 161/975 (16.5) | 1.216 (0.990-1.495) | 1.425 (1.141-1.779) |
| 8 | 20/127 (15.7) | 1.023 (0.625-1.672) | 1.582 (0.930-2.692) | 118/614 (19.2) | 1.463 (1.159-1.847) | 1.685 (1.309-2.170) |
| 9+10 | 12/67 (17.9) | 1.194 (0.632-2.254) | 1.421 (0.716-2.819) | 105/565 (18.6) | 1.404 (1.101-1.790) | 1.529 (1.175-1.988) |
| Shoulder Dystocia^§^ | | | | | | |
| 1+2 | 6/1211 (0.5) | 1.165 (0.414-3.280) | 1.158 (0.411-3.263) | 9/1475 (0.6) | 1.389 (0.563-3.427) | 1.355 (0.548-3.348) |
| 3 | 9/2114 (0.4) | 1.000 | 1.000 | 10/2273 (0.4) | 1.000 | 1.000 |
| 4 | 25/4729 (0.5) | 1.243 (0.579-2.668) | 1.252 (0.583-2.690) | 17/2877 (0.6) | 1.345 (0.615-2.943) | 1.332 (0.608-2.918) |
| 5 | 25/3135 (0.8) | 1.880 (0.876-4.036) | 1.910 (0.886-4.115) | 18/2391 (0.8) | 1.717 (0.791-3.727) | 1.729 (0.795-3.759) |
| 6 | 16/1154 (1.4) | 3.288 (1.449-7.465) | 3.276 (1.436-7.475) | 12/1693 (0.7) | 1.615 (0.696-3.748) | 1.631 (0.701-3.794) |
| 7 | 5/320 (1.6) | 3.713 (1.236-11.149) | 3.727 (1.229-11.296) | 7/971 (0.7) | 1.643 (0.624-4.330) | 1.635 (0.618-4.322) |
| 8 | 3/127 (2.4) | 5.659 (1.513-21.164) | 5.627 (1.497-21.152) | 4/613 (0.7) | 1.486 (0.465-4.756) | 1.470 (0.458-4.720) |
| 9+10 | 3/66 (4.5) | 11.138 (2.944-42.131) | 10.531 (2.777-39.941) | 15/563 (2.7) | 6.194 (2.768-13.863) | 5.959 (2.644-13.430) |
| Macrosomia^§^ | | | | | | |
| 1+2 | 19/1211 (1.6) | 0.895 (0.512-1.563) | 0.906 (0.518-1.586) | 39/1475 (2.6) | 0.908 (0.608-1.357) | 0.843 (0.562-1.264) |
| 3 | 37/2114 (1.8) | 1.000 | 1.000 | 66/2273 (2.9) | 1.000 | 1.000 |
| 4 | 117/4729 (2.5) | 1.424 (0.980-2.068) | 1.366 (0.939-1.988) | 75/2877 (2.6) | 0.895 (0.640-1.252) | 0.940 (0.670-1.318) |
| 5 | 112/3135 (3.6) | 2.080 (1.428-3.029) | 1.969 (1.347-2.876) | 70/2391 (2.9) | 1.009 (0.717-1.419) | 1.083 (0.767-1.528) |
| 6 | 55/1154 (4.8) | 2.809 (1.840-4.289) | 2.487 (1.622-3.815) | 58/1693 (3.4) | 1.186 (0.829-1.698) | 1.312 (0.913-1.887) |
| 7 | 16/320 (5.0) | 2.954 (1.624-5.376) | 2.643 (1.440-4.850) | 28/971 (2.9) | 0.993 (0.634-1.555) | 1.076 (0.684-1.694) |
| 8 | 9/127 (7.1) | 4.281 (2.019-9.079) | 3.532 (1.650-7.563) | 17/612 (2.8) | 0.954 (0.555-1.638) | 1.043 (0.604-1.799) |
| 9+10 | 9/66 (13.6) | 8.863 (4.085-19.230) | 8.062 (3.665-17.736) | 21/563 (3.7) | 1.296 (0.786-2.136) | 1.390 (0.838-2.306) |
| Large for Gestational Age Fetus^§^ | | | | | | |
| 1+2 | 76/1211 (6.3) | 0.786 (0.593-1.041) | 0.788 (0.593-1.046) | 134/1475 (9.1) | 0.875 (0.700-1.094) | 0.844 (0.673-1.059) |
| 3 | 166/2114 (7.9) | 1.000 | 1.000 | 233/2273 (10.3) | 1.000 | 1.000 |
| 4 | 436/4729 (9.2) | 1.192 (0.989-1.436) | 1.139 (0.944-1.376) | 274/2877 (9.5) | 0.922 (0.767-1.108) | 0.956 (0.793-1.152) |
| 5 | 368/3135 (11.7) | 1.561 (1.288-1.891) | 1.491 (1.227-1.813) | 253/2391 (10.6) | 1.036 (0.858-1.250) | 1.083 (0.895-1.311) |
| 6 | 170/1154 (14.7) | 2.027 (1.615-2.544) | 1.839 (1.459-2.317) | 195/1693 (11.5) | 1.140 (0.932-1.394) | 1.218 (0.992-1.496) |
| 7 | 57/320 (17.8) | 2.543 (1.833-3.528) | 2.372 (1.698-3.315) | 96/971 (9.9) | 0.961 (0.748-1.234) | 1.002 (0.777-1.292) |
| 8 | 31/127 (24.4) | 3.789 (2.453-5.853) | 3.240 (2.079-5.050) | 58/613 (9.5) | 0.915 (0.676-1.238) | 0.966 (0.711-1.313) |
| 9+10 | 21/66 (31.8) | 5.476 (3.186-9.413) | 5.198 (2.985-9.053) | 82/563 (14.6) | 1.493 (1.139-1.956) | 1.588 (1.205-2.093) |
| Low Birth Weight^§^ | | | | | | |
| 1+2 | 114/1211 (9.4) | 1.278 (0.993-1.643) | 1.287 (0.999-1.657) | 112/1475 (7.6) | 1.155 (0.896-1.488) | 1.150 (0.891-1.484) |
| 3 | 159/2114 (7.5) | 1.000 | 1.000 | 151/2273 (6.6) | 1.000 | 1.000 |
| 4 | 335/4729 (7.1) | 0.937 (0.770-1.141) | 0.966 (0.793-1.177) | 195/2877 (6.8) | 1.022 (0.820-1.273) | 1.014 (0.813-1.264) |
| 5 | 213/3135 (6.8) | 0.896 (0.724-1.109) | 0.922 (0.743-1.144) | 172/2391 (7.2) | 1.089 (0.868-1.366) | 1.087 (0.866-1.365) |
| 6 | 70/1154 (6.1) | 0.794 (0.594-1.062) | 0.849 (0.633-1.139) | 117/1693 (6.9) | 1.043 (0.812-1.340) | 1.037 (0.807-1.334) |
| 7 | 28/320 (8.8) | 1.179 (0.775-1.795) | 1.219 (0.798-1.863) | 79/971 (8.1) | 1.245 (0.938-1.652) | 1.252 (0.942-1.666) |
| 8 | 11/127 (8.7) | 1.166 (0.615-2.209) | 1.331 (0.700-2.531) | 53/613 (8.6) | 1.330 (0.960-1.844) | 1.314 (0.946-1.825) |
| 9+10 | 7/66 (10.6) | 1.459 (0.655-3.247) | 1.575 (0.705-3.521) | 58/563 (10.3) | 1.614 (1.174-2.218) | 1.576 (1.144-2.172) |
| Small for Gestational Age Fetus^§^ | | | | | | |
| 1+2 | 135/1211 (11.1) | 1.426 (1.124-1.808) | 1.434 (1.128-1.824) | 127/1475 (8.6) | 1.220 (0.958-1.553) | 1.212 (0.949-1.547) |
| 3 | 171/2114 (8.1) | 1.000 | 1.000 | 163/2273 (7.2) | 1.000 | 1.000 |
| 4 | 333/4729 (7.0) | 0.861 (0.710-1.043) | 0.899 (0.740-1.092) | 220/2877 (7.6) | 1.072 (0.868-1.323) | 1.088 (0.879-1.346) |
| 5 | 194/3135 (6.2) | 0.750 (0.605-0.928) | 0.785 (0.632-0.975) | 141/2391 (5.9) | 0.811 (0.643-1.024) | 0.819 (0.648-1.037) |
| 6 | 57/1154 (4.9) | 0.590 (0.434-0.804) | 0.659 (0.482-0.901) | 102/1693 (6.0) | 0.830 (0.643-1.072) | 0.841 (0.649-1.089) |
| 7 | 19/320 (5.9) | 0.717 (0.440-1.170) | 0.765 (0.467-1.254) | 71/971 (7.3) | 1.021 (0.765-1.364) | 1.070 (0.798-1.434) |
| 8 | 6/127 (4.7) | 0.563 (0.245-1.298) | 0.670 (0.289-1.552) | 52/613 (8.5) | 1.200 (0.866-1.662) | 1.239 (0.891-1.723) |
| 9+10 | 3/66 (4.5) | 0.541 (0.168-1.741) | 0.616 (0.190-1.998) | 42/563 (7.5) | 1.044 (0.734-1.484) | 1.078 (0.754-1.540) |
| Neonatal Hypoglycaemia^§^ | | | | | | |
| 1+2 | 46/1211 (3.8) | 1.375 (0.929-2.036) | 1.363 (0.920-2.019) | 49/1475 (3.3) | 1.149 (0.789-1.673) | 1.096 (0.752-1.597) |
| 3 | 59/2114 (2.8) | 1.000 | 1.000 | 66/2273 (2.9) | 1.000 | 1.000 |
| 4 | 144/4729 (3.0) | 1.094 (0.804-1.488) | 1.142 (0.839-1.555) | 91/2877 (3.2) | 1.092 (0.792-1.507) | 1.104 (0.799-1.525) |
| 5 | 89/3135 (2.8) | 1.018 (0.729-1.421) | 1.083 (0.774-1.516) | 50/2391 (2.1) | 0.714 (0.492-1.036) | 0.735 (0.506-1.068) |
| 6 | 40/1154 (3.5) | 1.251 (0.832-1.881) | 1.354 (0.897-2.043) | 55/1693 (3.2) | 1.123 (0.781-1.615) | 1.172 (0.814-1.689) |
| 7 | 10/320 (3.1) | 1.124 (0.569-2.220) | 1.185 (0.597-2.354) | 36/971 (3.7) | 1.288 (0.852-1.946) | 1.352 (0.892-2.048) |
| 8 | 10/127 (7.9) | 2.977 (1.485-5.969) | 3.322 (1.649-6.692) | 26/613 (4.2) | 1.481 (0.932-2.353) | 1.540 (0.967-2.451) |
| 9+10 | 6/66 (9.1) | 3.483 (1.447-8.382) | 3.623 (1.499-8.754) | 31/563 (5.5) | 1.949 (1.259-3.017) | 2.009 (1.294-3.119) |
| Respiratory Distress Syndrome^§^ | | | | | | |
| 1+2 | 5/1211 (0.4) | 0.970 (0.324-2.900) | 0.977 (0.326-2.925) | 5/1475 (0.3) | 0.770 (0.263-2.256) | 0.768 (0.262-2.256) |
| 3 | 9/2114 (0.4) | 1.000 | 1.000 | 10/2273 (0.4) | 1.000 | 1.000 |
| 4 | 25/4729 (0.5) | 1.243 (0.579-2.668) | 1.285 (0.597-2.763) | 7/2877 (0.2) | 0.552 (0.210-1.452) | 0.529 (0.201-1.393) |
| 5 | 10/3135 (0.3) | 0.748 (0.304-1.845) | 0.771 (0.311-1.910) | 14/2391 (0.6) | 1.333 (0.591-3.007) | 1.299 (0.575-2.936) |
| 6 | 6/1154 (0.5) | 1.222 (0.434-3.443) | 1.294 (0.456-3.669) | 5/1693 (0.3) | 0.670 (0.229-1.965) | 0.644 (0.219-1.893) |
| 7 | 3/320 (0.9) | 2.213 (0.596-8.220) | 2.275 (0.605-8.547) | 8/971 (0.8) | 1.880 (0.740-4.778) | 1.794 (0.703-4.578) |
| 8 | 2/127 (1.6) | 3.742 (0.800-17.504) | 4.359 (0.925-20.546) | 3/613 (0.5) | 1.113 (0.305-4.056) | 1.030 (0.281-3.768) |
| 9+10 | 0/66 (0.0) | NA^‡^ | NA^‡^ | 8/563 (1.4) | 3.262 (1.281-8.303) | 2.898 (1.129-7.436) |
| NICU Admission^§^ | | | | | | |
| 1+2 | 24/1211 (2.0) | 1.201 (0.711-2.029) | 1.200 (0.709-2.029) | 17/1475 (1.2) | 0.668 (0.376-1.185) | 0.659 (0.371-1.170) |
| 3 | 35/2114 (1.7) | 1.000 | 1.000 | 39/2273 (1.7) | 1.000 | 1.000 |
| 4 | 82/4729 (1.7) | 1.048 (0.703-1.562) | 1.096 (0.734-1.635) | 40/2877 (1.4) | 0.808 (0.518-1.260) | 0.778 (0.498-1.214) |
| 5 | 42/3135 (1.3) | 0.807 (0.513-1.268) | 0.855 (0.542-1.348) | 45/2391 (1.9) | 1.099 (0.713-1.694) | 1.080 (0.700-1.668) |
| 6 | 22/1154 (1.9) | 1.154 (0.674-1.977) | 1.259 (0.732-2.166) | 32/1693 (1.9) | 1.104 (0.689-1.769) | 1.078 (0.671-1.731) |
| 7 | 10/320 (3.1) | 1.916 (0.939-3.908) | 2.053 (0.999-4.220) | 18/971 (1.9) | 1.082 (0.616-1.901) | 1.045 (0.593-1.841) |
| 8 | 4/127 (3.1) | 1.932 (0.676-5.522) | 2.307 (0.803-6.630) | 11/613 (1.8) | 1.047 (0.533-2.056) | 0.987 (0.501-1.944) |
| 9+10 | 3/66 (4.5) | 2.829 (0.847-9.442) | 2.937 (0.875-9.863) | 20/563 (3.6) | 2.110 (1.221-3.646) | 1.939 (1.117-3.365) |
| †Adjusted for age, ethnicity, parity, gestational age at oral glucose tolerance test and baby’s sex. | | | | | | |
| *Miscarriages were excluded from the analysis | | | | | | |
| ^§^ Miscarriages and stillbirths were excluded from the analysis | | | | | | |
| ^‡^Regression analysis could not be conducted due to zero recorded cases of the complication in the corresponding glucose category | | | | | | |
